# Synthesis of isobemisiose, neosartose, and fischerose: three α-1,6-linked trehalose-based oligosaccharides identified from *Neosartorya fischeri*†

**DOI:** 10.1039/d0ra04137h

**Published:** 2020-06-12

**Authors:** E. J. Kuenstner, E. A. Palumbo, J. Levine, N. L. Snyder

**Affiliations:** Department of Chemistry, Davidson College Box 7120 Davidson NC 28036 USA nisnyder@davidson.edu

## Abstract

Three complex α-1,6-linked trehalose-based oligosaccharides with unique preservation properties, isobemisiose, neosartose, and fischerose, were recently identified from the extreme stress-tolerant ascospores of *Neosartorya fischeri.* Herein, we report the first concise, scalable, and iterative chemical synthesis of these oligosaccharides from a differentially protected thioglycoside donor and a selectively protected, asymmetric trehalose acceptor. This work constitutes an improved synthesis of isobemisiose, and is also the first reported synthesis of neosartose, a tetrasaccharide, and fischerose, a pentasaccharide, in good yield. Importantly, in-depth studies of biological function are enabled by this synthetic platform.

## Abstract 2

The first concise and scalable chemical synthesis of three complex α-1,6-linked trehalose-based oligosaccharides, isobemisiose, neosartose, and fischerose, are reported for the first time.

## Graphical abstract

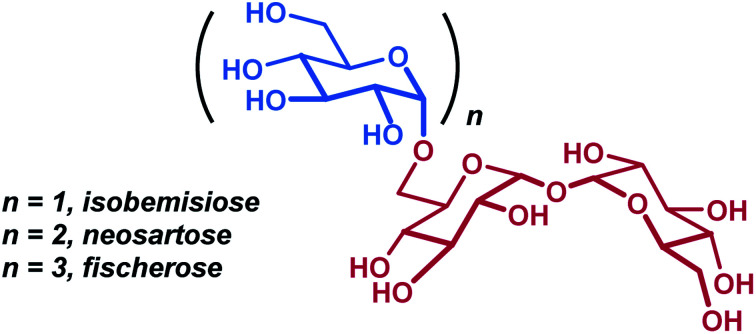

## Introduction

**Fig. 1:**
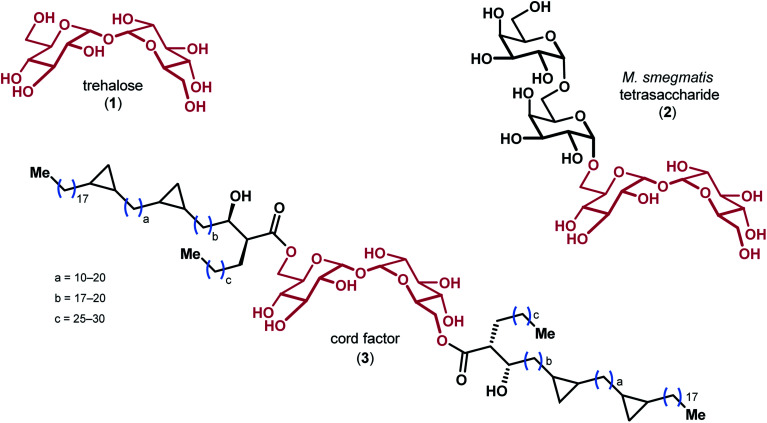
The structures of trehalose and representative derivatives of biological importance, with the trehalose core highlighted in red.

Trehalose 1, a disaccharide comprised of two glucose monomers linked in a α,α-1,1 fashion, is found in over 80 different species of plants, insects, algae, fungi, and bacteria (Fig. 1).^1^ The oligosaccharides and mycolic acids that contain trehalose (*e.g.*2 & 3) are important biomolecules involved in a variety of functions ranging from pathogenicity and autophagy induction to membrane stability.^2–4^ Notably, the atypical α,α-1,1 glycosidic linkage of trehalose renders the disaccharide non-reducing, which is believed to impart significant resistance to degradation by chemical, thermal, and desiccating means.^5,6^ The precise biophysical mechanism by which trehalose derivatives stabilize lipid membranes is the subject of ongoing investigation, and has previously been explained by a number of different hypotheses.^7–9^

**Fig. 2:**
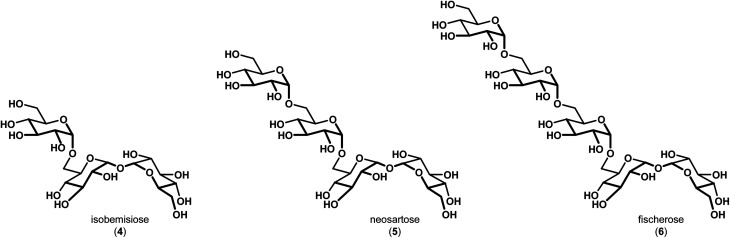
Trehalose-containing oligosaccharides with preservation capabilities identified in *Neosartorya fischeri* (*Aspergillus fischeri*).

In 2015, Wyatt *et al.* identified and characterized a series of trehalose-containing oligosaccharides responsible for the unique preservation properties in the extreme stress-resistant ascospores of *Neosartorya fischeri* (*Aspergillus fischeri*) (Fig. 2).^10–12^ FTIR spectroscopy was used to ascertain *T*_g_ and WTC values for combinations of isobemisiose 4, neosartose 5 and fischerose 6. In aggregate, the data suggested that the trehalose-containing oligosaccharides form a high-density glass that is postulated to serve a protective role for cytosolic biomolecules *in vivo*. Moreover, recent studies have indicated that sol gels embedded with bovine LDH have increased stability in the presence of neosartose 5 and fischerose 6 when compared to trehalose.^13^ The complexity of these compounds and their unique preservation properties ultimately led us to engage in a synthetic effort to prepare these compounds to supplement our ongoing studies to broaden our understanding of the role of these unique compounds in protein preservation. Our results are reported below.

## Results and discussion

**Fig. 3:**
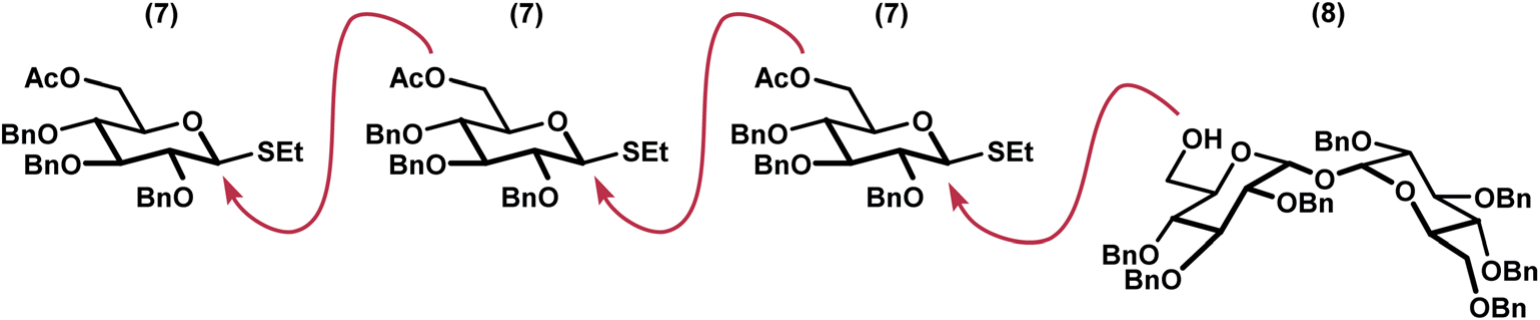
Iterative oligosaccharide synthesis by incorporation of a bifunctional glycosyl linchpin.

We envisioned a linear assembly of the oligosaccharides 4, 5, and 6*via* a series of iterative glycosylations. The crux of our strategy was the unification of a bifunctional glycosyl donor with an asymmetric trehalose acceptor. Whereas differential removal of the glucosyl C6 protecting group would allow for additional glycosylations, global deprotection would afford the requisite targets. These considerations led to the identification of readily accessible thioglycoside 7 (ref. 14) and trehalose derivative 8 (ref. 15) as suitable building blocks (Fig. 3). Thioglycoside 7 was particularly attractive because of the precedence for using this donor to generate α-glucosidic linkages with high diasteroselectivity, albeit with different nucleophiles, and for its straightforward synthesis on multigram scales.^14^ Both thioglycoside 7 and trehalose analog 8 were readily synthesized from commercially available building blocks.

**Scheme 1:**
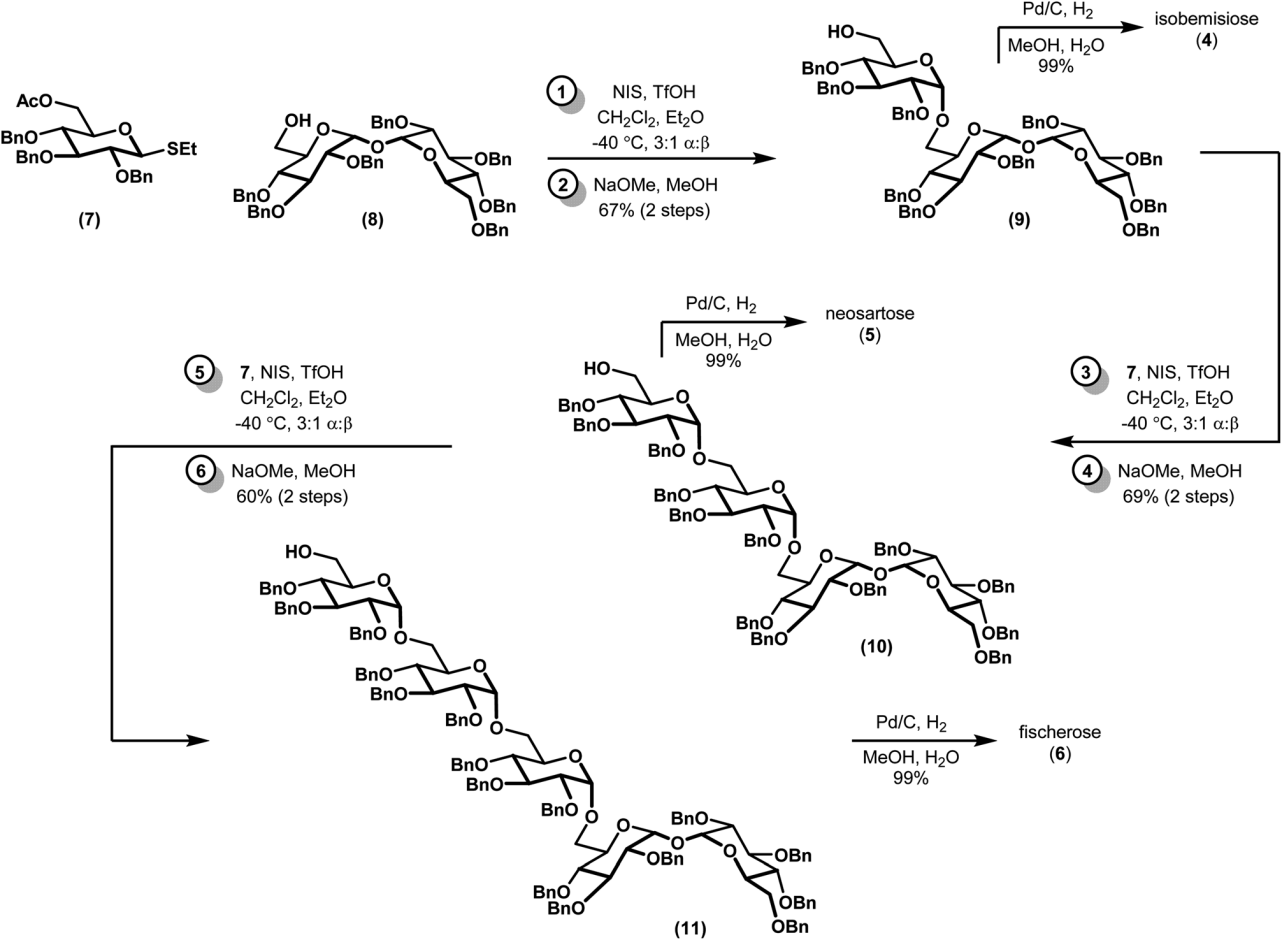
Synthesis of α-1,6-linked oligosaccharides.

Our synthesis began with a moderately stereoselective glycosylation of donor 7 with acceptor 8*via* the use of NIS and catalytic triflic acid to form trisaccharide 9 (Scheme 1). Despite the use of a non-participating benzyl group at C2 in conjunction with a binary solvent mixture, we were unable to achieve greater than 3 : 1 d.r. to favor the desired α anomer.^16,17^ Purification of these diastereomers was best achieved following saponification to alcohol 9 and the corresponding β-linked epimer (not shown). Tentative assignment of the relative stereochemistry of the newly-formed glycosidic bond was first accomplished by examination of anomeric resonances in ^13^C NMR, and later confirmed *via* comparison to a previously-synthesized sample of 9.^18^ Iterative glycosylations under identical conditions gave 10 and 11, which proceeded with comparable diastereoselectivity. Removal of the remaining benzyl protecting groups *via* hydrogenolysis required the addition of a few drops of water as a co-solvent to effect full dissolution of the partially deprotected oligosaccharides, but proceeded in near-quantitative yields. All ^13^C and ^1^H NMR spectra of the deprotected compounds, which had been presumed to be of the α configuration, fully matched the previous report, confirming our previous assignments of relative stereochemistry.^10^

The stereoselectivity of our glycosylation, while modest, merits additional discussion. Indeed, the reactivity of carbohydrate building blocks is critical for forming regio- and diastereoselective glycosidic linkages.^19–22^ First, our results with thioglycoside donor 7 differs from an earlier report by Koto and co-workers^18^ that remarkably predates the isolation of isobemisiose from a natural source. Their report, which involves glycosylation of acceptor 8 with the lactol (C1 hemiacetal) analog of 7 in the presence of excess pyridine and TMS triflate, gave *ca* 12 : 1 d.r. to favor the α-anomer albeit in lower yields and on smaller scales. While the original authors did not comment on the origin of this high diastereoselectivity, other reports have speculated that it may arise *via* remote participation of the C6 acetyl group.^23^ Indeed, similar mechanisms of selectivity have been noted in the synthesis of an epimeric trehalose-containing galactoside.^24^ However, the presence of a C6 acetate in 7 suggests a more complex interplay between remote participation and various ion-pair species. Recent reports by Zhu^25^ and Kowalska,^26^ which were published while this manuscript was in preparation, suggest that the α-selectivity of this reaction might be improved by using trifluoroacetimidates instead of thioglycoside donors. In contrast, the role of glycosylation acceptors in promoting stereochemical outcomes is less established than that of glycosylation donors, but research in this area may still provide relevant insight to our results. Numerous studies across various reaction manifolds have correlated reactive acceptors (*e.g.* primary alcohols) with β-selectivity.^27,28^ Conversely, less reactive counterparts (*e.g.* secondary alcohols with adjacent electron-withdrawing groups) generally display α-selectivity.^22^ It might be expected, then, that replacement of the benzyl protecting groups in 8 for benzoyl groups could improve the diastereoselectivity of our glycosylation. These speculations aside, the present paper emphasizes the impact of seemingly subtle changes in stereoelectronic environments during the formation of reactive intermediates in glycosylations.

In conclusion, we have described a concise synthesis of three complex oligosaccharides: isobemisiose (4), neosartose (5), and fischerose (6). Our sequence to 6, which is the largest synthesized α-linked trehalose-derived oligosaccharide described to date, is unprecedented. Sequences to the former species compare favorably to reports of enzymatic incubations and *de novo* syntheses of smaller trehalose oligosaccharides.^18,24,29–33^ Several advantages over the single study for isobemisiose reported by Koto^18^ are particularly noteworthy, including the overall yield and scalability (*e.g.* 66% of 4 over three steps on a gram scale, as opposed to *ca*. 45% over three steps on a hundred milligram scale). In addition, our methodology provides us with the ability to generate theretofore unknown stereoisomers. The aforementioned compounds are the subject of ongoing biological investigations in our laboratory, the results of which will be reported in due course.

## Conflicts of interest

There are no conflicts of interest to declare.

## Supplementary Material

RA-010-D0RA04137H-s001
