# Supplementary material for: Synthesis of isobemisiose, neosartose, and fischerose: three α-1,6-linked trehalose-based oligosaccharides identified from Neosartorya fischeri
Source: RSC Adv. 2020 Jun 12;10(38):22726–9. doi: 10.1039/d0ra04137h (PMC9054618; doi:10.1039/d0ra04137h)
Supplement: RA-010-D0RA04137H-s001 [file RA-010-D0RA04137H-s001.pdf]

Electronic Supplementary Material (ESI) for RSC Advances.
This journal is © The Royal Society of Chemistry 2020

Supplemental Data

"Synthesis of Isobemisiose, Neosartose, and Fischerose: Three α-1,6-Linked Trehalose-Based Oligosaccharides Identified from *Neosartorya fischeri*"

Kuenstner, E.J.; Palumbo, E.A.; Levine, J.; Snyder, N.L.

Contents:

Materials and Methods **Page 2**

General Glycosylation Procedure **Page 2**

General Hydrogenolysis Procedure **Page 3**

Characterization Data **Pages 4 - 13**

^{1}H and ^{13}C NMR Spectra **Pages 14 - 32**

Materials and Methods

All reactions were conducted in oven-dried glassware under positive pressure of dry nitrogen gas unless indicated otherwise. Reaction solvents were dried via percolation through a column packed with neutral alumina under positive pressure of dry argon gas. NMR solvents were purchased from Cambridge Isotope Laboratories and used without further purification. NIS was freshly recrystallized from dioxane and carbon tetrachloride prior to use. All other reagents were used without further purification. Reactions were monitored with TLC using precoated silica gel plates (Merck silica gel 60, F_{254}) and the plates were visualized with vanillin stain. Flash chromatography was performed using silica gel (Merck 60 Å, 230–400 mesh, 32–63 μm). ^{13}C and ^{1}H NMR spectra were recorded on a Bruker Avance 500 MHz instrument with a BBO probe, and HRMS were obtained by the Mass Spectroscopy Center at the University of South Carolina with special thanks to Dr Michael Walla and Dr. William Cotham for HRMS support.

General Glycosylation Procedure

The requisite acceptor (1.0 equiv.) and thioglycoside **7** (1.2 equiv.) were dissolved in Et_2O and CH_2Cl_2 (0.05 M, 5:1). Activated 3Å molecular sieves were then added. After 30 minutes of stirring at ambient temperature, the mixture was cooled to -40 °C and NIS (2.0 equiv.) was added in a single portion, followed by the dropwise addition of TfOH (0.5 equiv.). The reaction was allowed to proceed until the acceptor was completely consumed (*ca.* 30 minutes) as judged by TLC (30% v/v EtOAc in hexanes), at which point it was then diluted with CH_2Cl_2 and quenched with saturated $NaHCO_3$. The biphasic mixture was separated, and the organic portion was washed successively with saturated $Na_2S_2O_3$ and saturated NaCl, then dried with anhydrous Na_2SO_4 and concentrated. The residue was dissolved in a minimal amount of EtOAc in hexanes (30% v/v) and passed through a short plug of silica to remove any unreacted

reagents and hydrolyzed **7**. Fractions containing an inseparable mixture of the anomers (~3:1 ratio by NMR) were combined and concentrated *in vacuo* to give a clear oil. The mixture of fully protected oligosaccharides was subsequently dissolved in methanolic NaOMe (0.1 M, 2 mg NaOMe/mL MeOH). Upon completion of the reaction (*ca.* 30 min), the solution was neutralized with the addition of Amberlite® IR 120H to achieve a pH between 7 and 8 and concentrated *in vacuo*. The resulting residue was purified with flash column chromatography (elution with 30% v/v EtOAc in hexanes) to give the pure alpha anomer as a clear and colorless oil.

General Hydrogenolysis Procedure

Saponified oligomers were dissolved in a minimal amount of MeOH (approximately 100 mL/g) and treated with approximately 250-500 mL of hydrogen and a catalytic amount of Pd/C. The reaction was allowed to proceed until complete as monitored by TLC. For larger oligomers, small quantities of water were added to the reaction to solubilize partially hydrogenated oligomers. The completed reactions were flooded with nitrogen to remove excess hydrogen gas and filtered over a bed of Celite. The Celite was rinsed repeatedly with MeOH and water (when necessary) to ensure complete recovery of each oligosaccharide. The solution was concentrated and filtered through a syringe filter prior to analysis.

Characterization Data

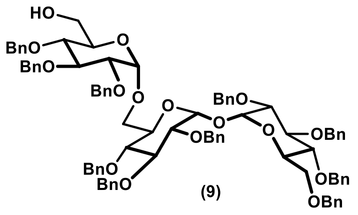

^{1}H NMR (500 MHz, $CDCl_3$): δ 7.44 – 7.28 (m, 35H), 7.29 – 7.19 (m, 13H), 7.18 – 7.14 (m, 2H), 5.26 (d, *J* = 3.6 Hz, 1H), 5.19 (d, *J* = 3.6 Hz, 1H), 5.06 – 4.82 (m, 9H), 4.81 – 4.55 (m, 10H), 4.49 (d, *J* = 10.7 Hz, 1H), 4.41 (d, *J* = 12.1 Hz, 1H), 4.19 (tt, *J* = 10.3, 2.7 Hz, 2H), 4.07 (td, *J* = 9.3, 5.9 Hz, 2H), 3.97 (t, *J* = 9.2 Hz, 1H), 3.83 (t, *J* = 9.6 Hz, 1H), 3.76 – 3.61 (m, 6H), 3.57 – 3.42 (m, 5H), 3.39 (dd, *J* = 10.7, 2.1 Hz, 1H), 1.62 (dd, *J* = 7.7, 5.2 Hz, 1H).

^{13}C NMR (125 MHz, $CDCl_3$): δ 139.05, 139.02, 138.89, 138.67, 138.55, 138.52, 138.43, 138.39, 138.30, 137.98, 128.54, 128.52, 128.48, 128.45, 128.43, 128.42, 128.11, 128.10, 128.06, 128.03, 128.02, 128.00, 127.89, 127.80, 127.78, 127.75, 127.74, 127.68, 127.62, 127.60, 127.55, 127.40, 97.48, 94.37, 94.31, 81.88, 81.82, 81.63, 80.31, 79.89, 79.58, 77.83, 77.78, 77.48, 77.42, 77.17, 76.91, 75.70, 75.65, 75.56, 75.16, 75.06, 73.64, 73.00, 72.30, 71.37, 70.95, 70.76, 68.33, 65.73, 62.02.

HRMS (ESI) $C_{88}H_{92}O_{16}$ $[M+NH_4]^+$ Calculated: 1422.6729 Found: 1422.6732.

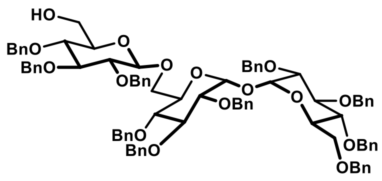

(SI-1)

^{1}H NMR (500 MHz, $CDCl_3$): δ 7.53 – 7.28 (m, 41H), 7.25 – 7.14 (m, 9H), 5.30 – 5.24 (m, 2H), 5.05 – 4.79 (m, 10H), 4.76 – 4.61 (m, 5H), 4.59 (d, *J* = 12.1 Hz, 1H), 4.51 (d, *J* = 10.8 Hz, 2H), 4.42 (d, *J* = 12.1 Hz, 1H), 4.28 (d, *J* = 7.8 Hz, 1H), 4.21 (d, *J* = 10.1 Hz, 2H), 4.07 (td, *J* = 9.3, 6.3 Hz, 2H), 3.91 – 3.80 (m, 2H), 3.78 – 3.39 (m, 10H), 3.33 (ddd, *J* = 9.7, 4.7, 2.7 Hz, 1H), 2.00 (t, *J* = 10.0 Hz, 1H).

^{13}C NMR (125 MHz, $CDCl_3$): δ 139.03, 138.95, 138.64, 138.50, 138.46, 138.37, 138.26, 138.24, 138.09, 137.97, 129.01, 128.99, 128.58, 128.54, 128.48, 128.46, 128.44, 128.42, 128.38, 128.10, 128.06, 128.00, 127.98, 127.96, 127.78, 127.76, 127.71, 127.67, 127.64, 127.61, 127.54, 127.51, 127.45, 103.72, 94.61, 94.52, 84.76, 81.94, 81.88, 81.74, 79.70, 79.41, 77.83, 77.71, 77.41, 77.16, 76.90, 75.78, 75.67, 75.59, 75.26, 75.17, 75.13, 75.06, 74.90, 73.60, 72.92, 72.77, 70.83, 70.08, 68.28, 68.18, 62.11.

HRMS (ESI) $C_{88}H_{92}O_{16}$ $[M+NH_4]^+$ Calculated: 1422.6729 Found: 1422.6777.

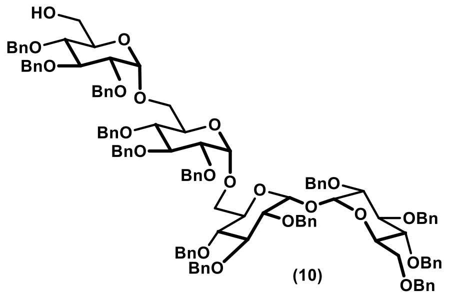

(10)

^{1}H NMR (500 MHz, $CDCl_3$): δ 7.37 – 7.27 (m, 48H), 7.26 – 7.23 (m, 7H), 7.23 – 7.18 (m, 8H), 7.16 – 7.13 (m, 2H), 5.27 (d, *J* = 3.6 Hz, 1H), 5.19 (d, *J* = 3.6 Hz, 1H), 5.07 (d, *J* = 3.5 Hz, 1H), 5.01 – 4.97 (m, 2H), 4.96 – 4.91 (m, 4H), 4.89 – 4.87 (m, 2H), 4.86 – 4.85 (m, 1H), 4.81 (dd, *J* = 15.4, 10.8 Hz, 2H), 4.75 – 4.60 (m, 9H), 4.59 – 4.47 (m, 6H), 4.40 (d, *J* = 12.1 Hz, 1H), 4.21 (dd, *J* = 10.0, 2.0 Hz, 1H), 4.17 (dt, *J* = 10.2, 2.8 Hz, 1H), 4.05 (q, *J* = 9.5 Hz, 2H), 4.00 (t, *J* = 9.2 Hz, 1H), 3.94 (t, *J* = 9.2 Hz, 1H), 3.85 – 3.78 (m, 3H), 3.75 – 3.68 (m, 4H), 3.67 – 3.59 (m, 4H), 3.56 – 3.48 (m, 4H), 3.46 – 3.37 (m, 3H), 1.63 – 1.58 (m, 1H).

^{13}C NMR (125 MHz, $CDCl_3$): δ 139.05, 138.90, 138.79, 138.72, 138.67, 138.55, 138.43, 138.40, 138.31, 137.99, 128.55, 128.51, 128.49, 128.46, 128.44, 128.42, 128.37, 128.14, 128.11, 128.05, 128.03, 128.01, 127.94, 127.90, 127.85, 127.80, 127.76, 127.73, 127.69, 127.68, 127.65, 127.60, 127.58, 127.54, 127.48, 127.40, 97.58, 97.31, 94.20, 94.15, 81.85, 81.81, 81.76, 81.63, 80.53, 80.30, 79.90, 79.49, 77.86, 77.82, 77.52, 77.49, 75.67, 75.63, 75.61, 75.48, 75.14, 75.12, 75.10, 75.06, 73.64, 73.01, 72.95, 72.40, 72.31, 71.44, 71.03, 70.90, 70.74, 68.37, 65.91, 65.56, 62.02.

HRMS (ESI) $C_{115}H_{120}O_{21}$ $[M+NH_4]^+$ Calculated: 1854.8666 Found: 1854.8616.

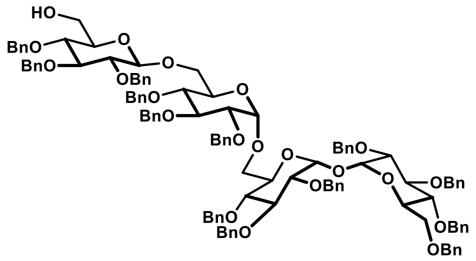

(SI-2)

^{1}H NMR (500 MHz, $CDCl_3$): δ 7.64 – 7.07 (m, 65H), 5.40 (d, *J* = 3.6 Hz, 1H), 5.36 (d, *J* = 3.6 Hz, 1H), 5.22 (d, *J* = 3.5 Hz, 1H), 5.19 – 4.97 (m, 11H), 4.95 – 4.80 (m, 7H), 4.80 – 4.68 (m, 5H), 4.65 (t, *J* = 10.9 Hz, 2H), 4.56 (d, *J* = 12.1 Hz, 1H), 4.50 (d, *J* = 7.7 Hz, 1H), 4.41 – 4.31 (m, 2H), 4.29 – 4.18 (m, 3H), 4.12 (t, *J* = 9.2 Hz, 1H), 4.07 – 3.88 (m, 4H), 3.90 – 3.78 (m, 4H), 3.78 – 3.60 (s, 8H), 3.62 – 3.52 (m, 1H), 3.49 (ddd, *J* = 9.6, 4.5, 2.7 Hz, 1H). 2.22 – 2.17 (m, 1H).

^{13}C NMR (125 MHz, $CDCl_3$): δ 138.95, 138.91, 138.64, 138.62, 138.50, 138.48, 138.46, 138.29, 138.22, 138.17, 138.05, 137.90, 128.94, 128.92, 128.51, 128.41, 128.35, 128.33, 128.31, 128.26, 128.04, 128.01, 127.99, 127.95, 127.93, 127.91, 127.88, 127.82, 127.79, 127.70, 127.67, 127.62, 127.57, 127.55, 127.53, 127.51, 127.45, 127.31, 103.84, 97.48, 93.98, 84.64, 82.00, 81.71, 81.56, 80.05, 79.78, 79.33, 77.73, 77.64, 77.42, 77.16, 76.91, 75.68, 75.56, 75.54, 75.37, 75.21, 75.06, 75.03, 75.00, 74.84, 74.80, 73.54, 72.98, 72.89, 72.70, 72.19, 71.31, 70.66, 69.84, 68.61, 68.29, 65.91, 62.02.

HRMS (ESI) $C_{115}H_{120}O_{21}$ $[M+NH_4]^+$ Calculated: 1854.8666 Found: 1854.8602.

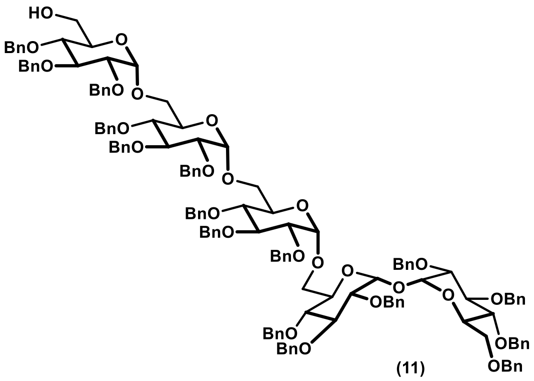

(11)

^{1}H NMR (500 MHz, $CDCl_3$): δ 7.34 – 7.30 (m, 12H), 7.29 – 7.23 (m, 48H), 7.22 – 7.15 (m, 18H), 7.13 – 7.10 (m, 2H), 5.23 (d, *J* = 3.6 Hz, 1H), 5.16 (d, *J* = 3.6 Hz, 1H), 5.02 (d, *J* = 3.5 Hz, 1H), 5.00 (d, *J* = 3.5 Hz, 1H), 4.96 – 4.90 (m, 8H), 4.88 – 4.81 (m, 6H), 4.76 (t, *J* = 11.2 Hz, 2H), 4.70 – 4.64 (m, 7H), 4.62 – 4.56 (m, 3H), 4.54 – 4.50 (m, 4H), 4.45 (d, *J* = 11.5 Hz, 2H), 4.37 (d, *J* = 12.1 Hz, 1H), 4.18 (d, *J* = 9.4 Hz, 1H), 4.14 (dt, *J* = 10.2, 2.7 Hz, 1H), 4.06 – 4.00 (m, 2H), 3.96 (td, *J* = 9.2, 3.9 Hz, 2H), 3.91 (t, *J* = 9.2 Hz, 1H), 3.84 – 3.76 (m, 5H), 3.74 – 3.63 (m, 6H), 3.63 – 3.56 (m, 4H), 3.54 – 3.44 (m, 4H), 3.40 (dt, *J* = 9.6, 3.5 Hz, 2H), 3.38 – 3.33 (m, 2H), 1.57 (dd, *J* = 7.5, 5.1 Hz, 1H).

^{13}C NMR (125 MHz, $CDCl_3$): δ 139.03, 139.02, 138.99, 138.86, 138.81, 138.75, 138.71, 138.66, 138.51, 138.49, 138.40, 138.38, 138.28, 137.96, 128.50, 128.46, 128.43, 128.41, 128.39, 128.38, 128.36, 128.33, 128.12, 128.10, 128.07, 128.03, 128.01, 127.99, 127.98, 127.91, 127.90, 127.85, 127.82, 127.76, 127.74, 127.72, 127.69, 127.63, 127.61, 127.57, 127.55, 127.52, 127.49, 127.47, 127.41, 127.35, 97.55, 97.20, 94.16, 81.81, 81.77, 81.73, 81.70, 81.56, 80.52, 80.48, 80.27, 79.90, 79.50, 77.80, 77.50, 77.41, 77.15, 76.90, 75.63, 75.59, 75.56, 75.52, 75.43, 75.09, 75.07, 75.04, 74.99, 73.60, 72.98, 72.94, 72.33, 72.30, 71.47, 71.00, 70.98, 70.95, 70.70, 68.34, 65.72, 65.50, 61.95.

HRMS (ESI) $C_{142}H_{148}O_{26}$ $[M+H]^+$ Calculated: 2270.0337 Found: 2270.0283.

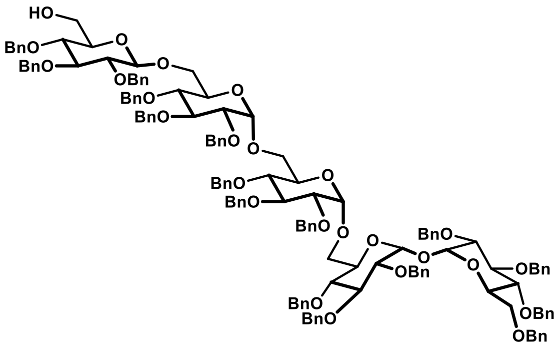

(SI-3)

^{1}H NMR (500 MHz, $CDCl_3$): δ 7.54 – 7.15 (m, 80H), 5.36 (d, *J* = 3.6 Hz, 1H), 5.28 (d, *J* = 3.6 Hz, 1H), 5.21 (d, *J* = 3.5 Hz, 1H), 5.11 – 5.00 (m, 8H), 4.98 – 4.89 (m, 6H), 4.89 – 4.71 (m, 11H), 4.71 – 4.61 (m, 4H), 4.61 – 4.54 (m, 3H), 4.49 (d, *J* = 12.1 Hz, 1H), 4.41 (d, *J* = 7.7 Hz, 1H), 4.32 – 4.24 (m, 2H), 4.19 – 4.00 (m, 5H), 3.98 – 3.84 (m, 5H), 3.81 – 3.69 (m, 8H), 3.66 – 3.56 (m, 4H), 3.54 – 3.44 (m, 3H), 3.42 – 3.39 (m, 1H), 2.08 (dd, *J* = 7.8, 5.8 Hz, 1H).

^{13}C NMR (125 MHz, $CDCl_3$): δ 139.05, 139.03, 138.98, 138.82, 138.70, 138.68, 138.64, 138.56, 138.55, 138.52, 138.39, 138.30, 138.25, 138.10, 137.97, 129.00, 128.57, 128.47, 128.46, 128.42, 128.40, 128.37, 128.33, 128.11, 128.09, 128.07, 128.01, 127.98, 127.95, 127.93, 127.84, 127.75, 127.72, 127.70, 127.65, 127.63, 127.60, 127.56, 127.53, 127.52, 127.50, 127.48, 127.41, 127.35, 103.89, 97.50, 97.45, 94.08, 84.70, 82.03, 81.81, 81.75, 81.73, 81.61, 80.50, 80.09, 79.90, 79.45, 77.82, 77.79, 77.72, 77.69, 77.42, 77.16, 76.91, 75.76, 75.63, 75.59, 75.50, 75.43, 75.25, 75.12, 75.09, 75.03, 74.96, 74.88, 73.60, 72.98, 72.89, 72.28, 72.20, 71.50, 70.86, 70.69, 69.94, 68.60, 68.35, 65.74, 65.67, 62.07.

HRMS (ESI) $C_{142}H_{148}O_{26}$ $[M+H]^+$ Calculated: 2270.0337 Found: 2270.0239.

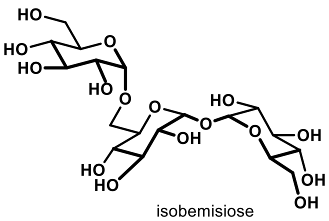

isobemisiose
(**4**)

^{1}H NMR (500 MHz, D_2O): δ 5.23 (apparent d, *J* = 3.9 Hz, 2H), 5.00 (d, *J* = 3.7 Hz, 1H), 4.07–3.98 (m, 2H), 3.91–3.84 (m, 5H), 3.82–3.73 (m, 5H), 3.72–3.66 (m, 2H), 3.62–3.56 (m, 2H), 3.49–3.35 (m, 2H).

^{13}C NMR (125 MHz, D_2O): δ 98.20, 93.92, 93.86, 73.61, 73.33, 73.05, 72.73, 72.34, 71.96, 71.52, 71.49, 71.05, 70.18, 70.16, 70.03, 65.89, 61.03, 61.00.

HRMS (ESI) $C_{18}H_{32}O_{16}$ $[M+Na]^+$ Calculated: 527.1588 Found: 527.1589.

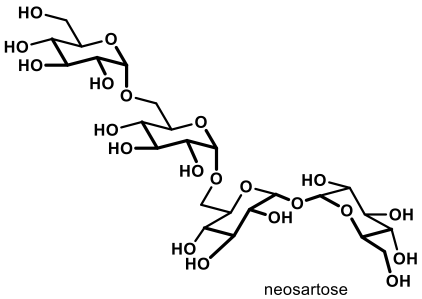

neosartose
(**5**)

^{1}H NMR (500 MHz, D_2O): δ 5.15–5.13 (m, 2H), 4.93–4.89 (m, 2H), 4.00–3.82 (m, 4H), 3.81–3.74 (m, 5H), 3.73–3.63 (m, 7H), 3.63–3.57 (m, 2H), 3.56–3.42 (m, 4H), 3.41–3.35(m, 2H).

^{13}C NMR (125 MHz, D_2O): δ 98.20, 98.10, 93.87, 93.80, 73.83, 73.56, 73.31, 73.01, 72.68, 72.29, 71.95, 71.84, 71.47, 71.44, 70.95, 70.71, 70.15, 70.13, 70.00, 69.98, 66.00, 65.90, 60.98, 60.95.

HRMS (ESI) $C_{24}H_{42}O_{21}$ $[M+Na]^+$ Calculated: 689.2116 Found: 689.2109.

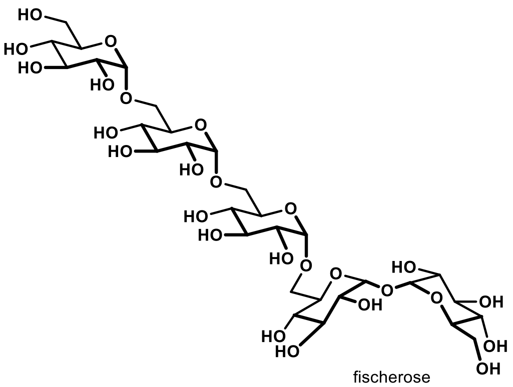

fischerose
(**6**)

^{1}H NMR (500 MHz, D_2O): δ 5.23–5.17 (m, 2H), 5.00–4.95 (m, 3H), 4.05–3.95 (m, 4H), 3.95–3.88 (m, 2H), 3.88–3.80 (m, 5H), 3.80–3.69 (m, 9H), 3.69–3.63 (m, 2H), 3.61–3.49 (m, 6H), 3.48–3.40 (m, 2H).

^{13}C NMR (125 MHz, D_2O): δ 98.20, 98.14, 98.09, 93.86, 93.80, 73.85, 73.82, 73.55, 73.30, 73.00, 72.68, 72.28, 71.94, 71.85, 71.83, 71.46, 71.43, 70.93, 70.71, 70.64, 70.14, 70.13, 70.03, 69.97, 66.07, 66.00, 65.87, 60.97, 60.94.

HRMS (ESI) $C_{30}H_{52}O_{26}$ $[M+Na]^+$ Calculated: 851.2645 Found: 851.2655.

^{1}H and ^{13}C NMR Spectra

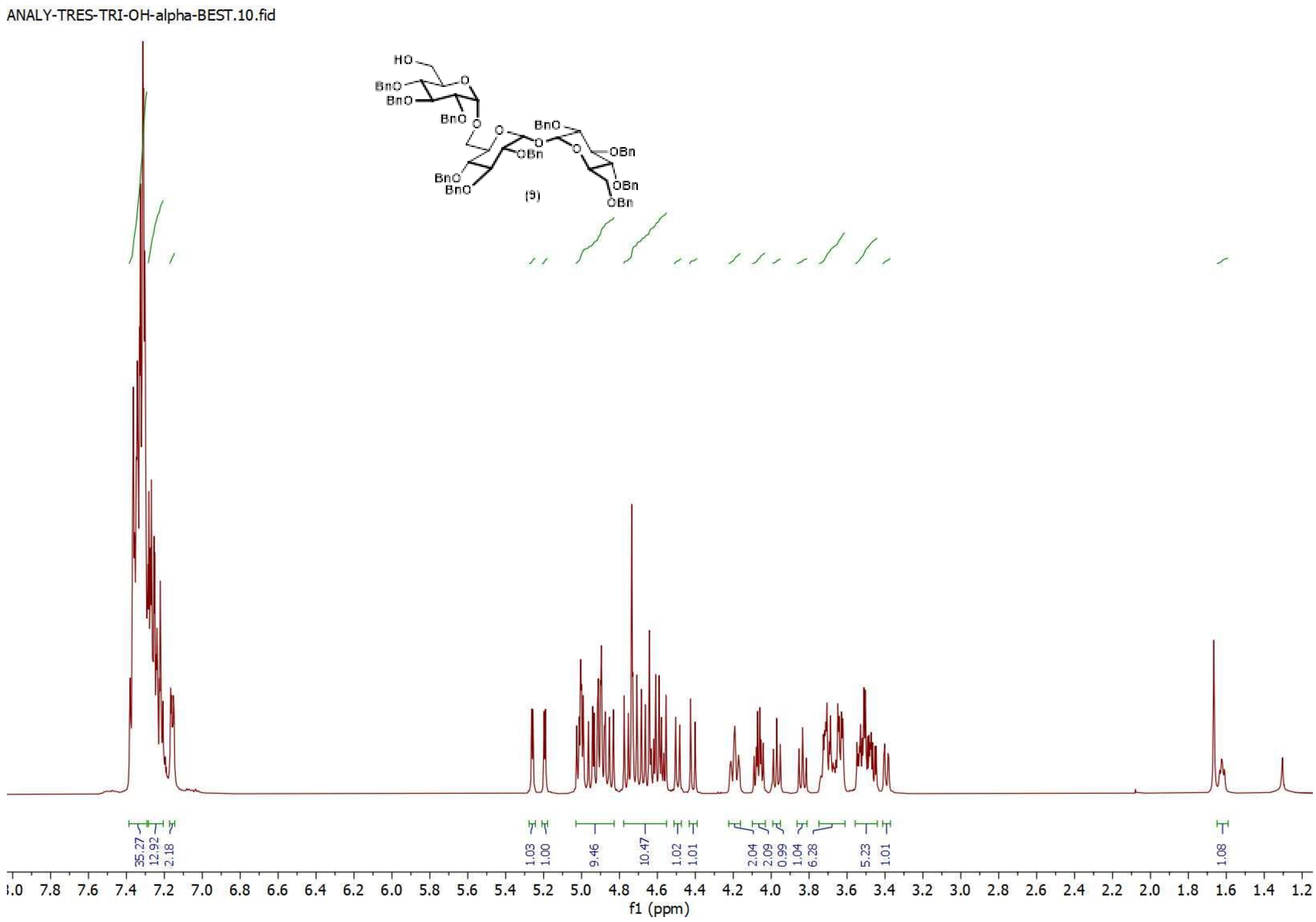

ANALY-TRES-TRI-OH-alpha-BEST.10.fid
HO
BnO
BnO
BnO
BnO
OBn
BnO
BnO
OBn
OBn
OBn
(9)
35.27
12.92
2.18
1.03
1.00
9.46
10.47
1.02
1.01
2.04
2.09
0.99
1.04
6.28
5.23
1.01
1.08
f1 (ppm)

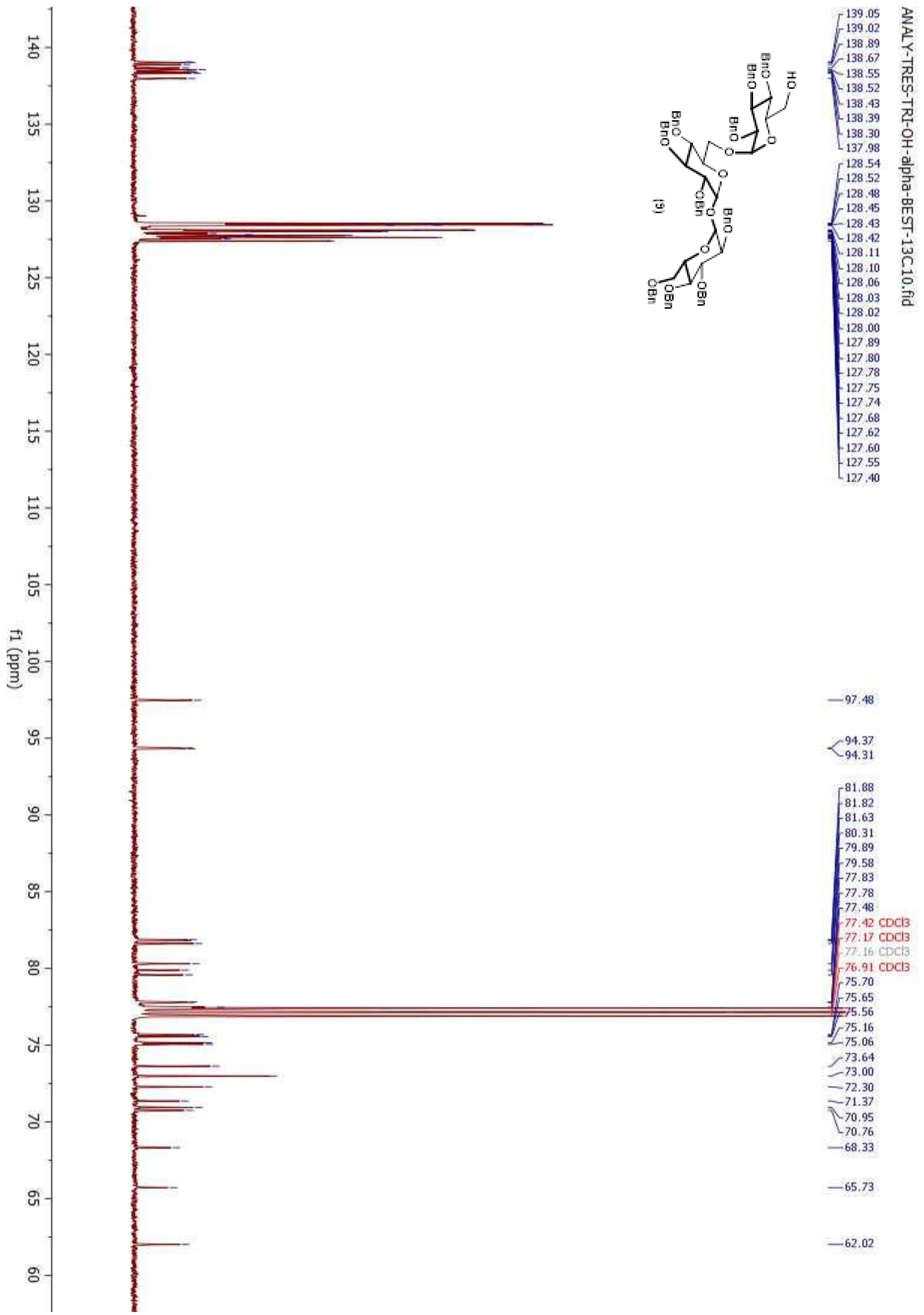

ANALY-TRES-TRI-OH-alpha-BEST-13C.10.fid
139.05
139.02
138.89
138.67
138.55
138.52
138.43
138.39
138.30
137.98
128.54
128.52
128.48
128.45
128.43
128.42
128.11
128.10
128.06
128.03
128.02
128.00
127.89
127.80
127.78
127.75
127.74
127.68
127.62
127.60
127.55
127.40
97.48
94.37
94.31
81.88
81.82
81.63
80.31
79.89
79.58
77.83
77.78
77.48
77.42 CDCl3
77.17 CDCl3
77.16 CDCl3
76.91 CDCl3
75.70
75.65
75.56
75.16
75.06
73.64
73.00
72.30
71.37
70.95
70.76
68.33
65.73
62.02
HO
BnO
OBn
(9)
f1 (ppm)

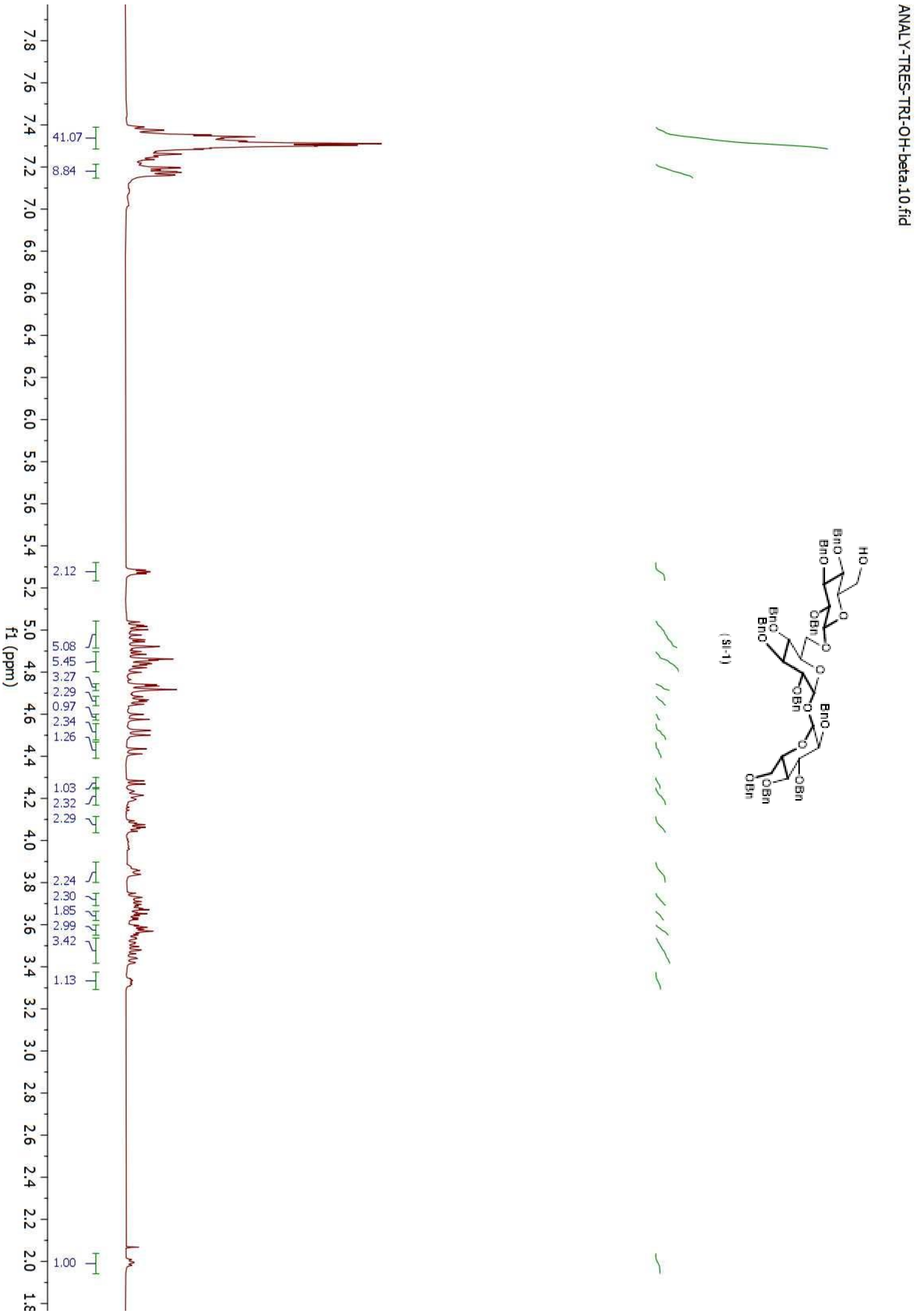

ANALY-TRES-TRI-OH-beta.10.fid
(SI-1)
f1 (ppm)

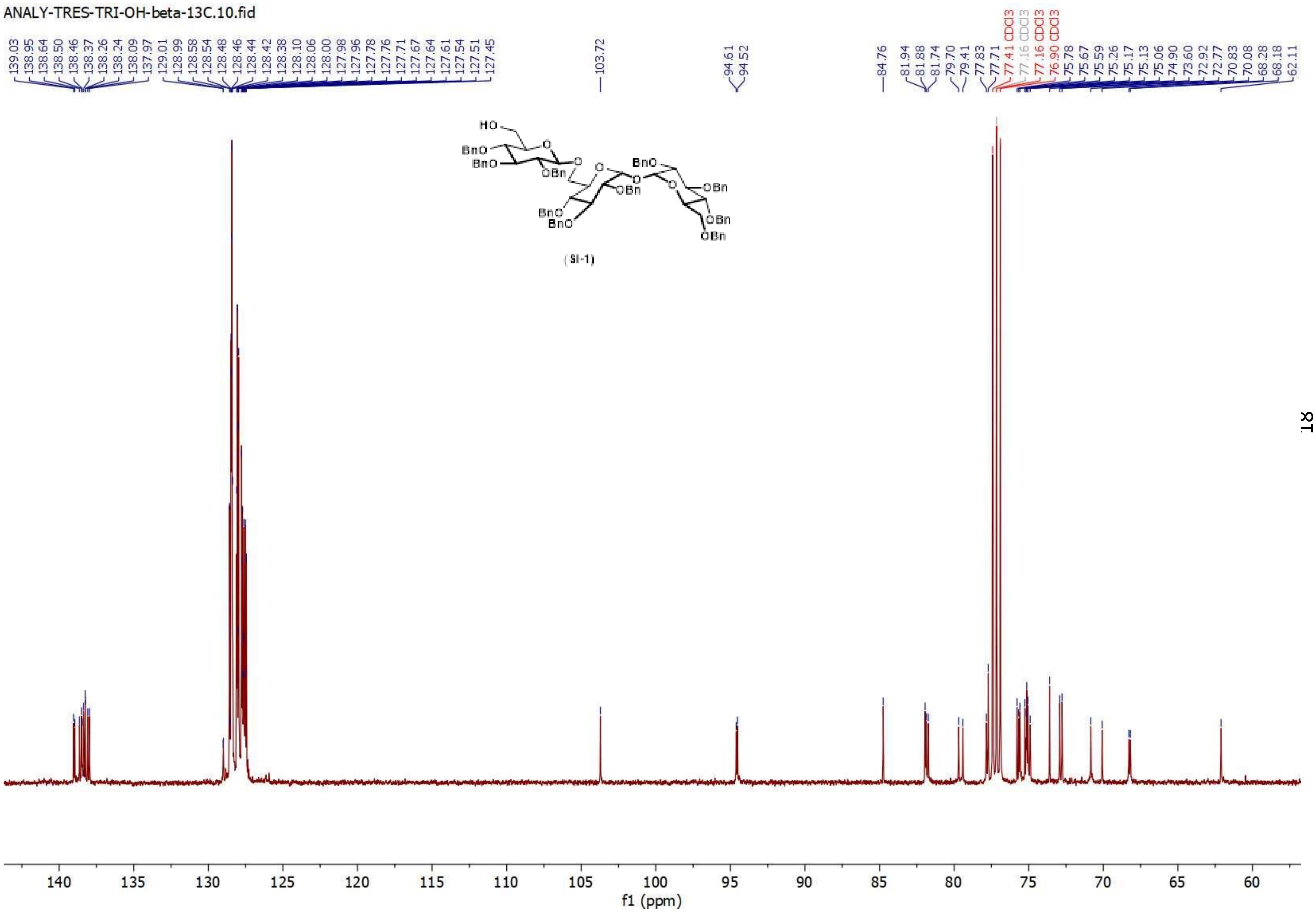
ANALY-TRES-TRI-OH-beta-13C.10.fid
139.03
138.95
138.64
138.50
138.46
138.37
138.26
138.24
138.09
137.97
129.01
128.99
128.58
128.54
128.48
128.46
128.44
128.42
128.38
128.10
128.06
128.00
127.98
127.96
127.78
127.76
127.71
127.67
127.64
127.61
127.54
127.51
127.45
103.72
94.61
94.52
84.76
81.94
81.88
81.74
79.70
79.41
77.83
77.71
77.41 CDCl3
77.16 CDCl3
77.16 CDCl3
76.90 CDCl3
75.78
75.67
75.59
75.26
75.17
75.13
75.06
74.90
73.60
72.92
72.77
70.83
70.08
68.28
68.18
62.11
HO
BnO
BnO
OBn
BnO
BnO
OBn
BnO
OBn
OBn
OBn
(SI-1)
140
135
130
125
120
115
110
105
100
95
90
85
80
75
70
65
60
f1 (ppm)

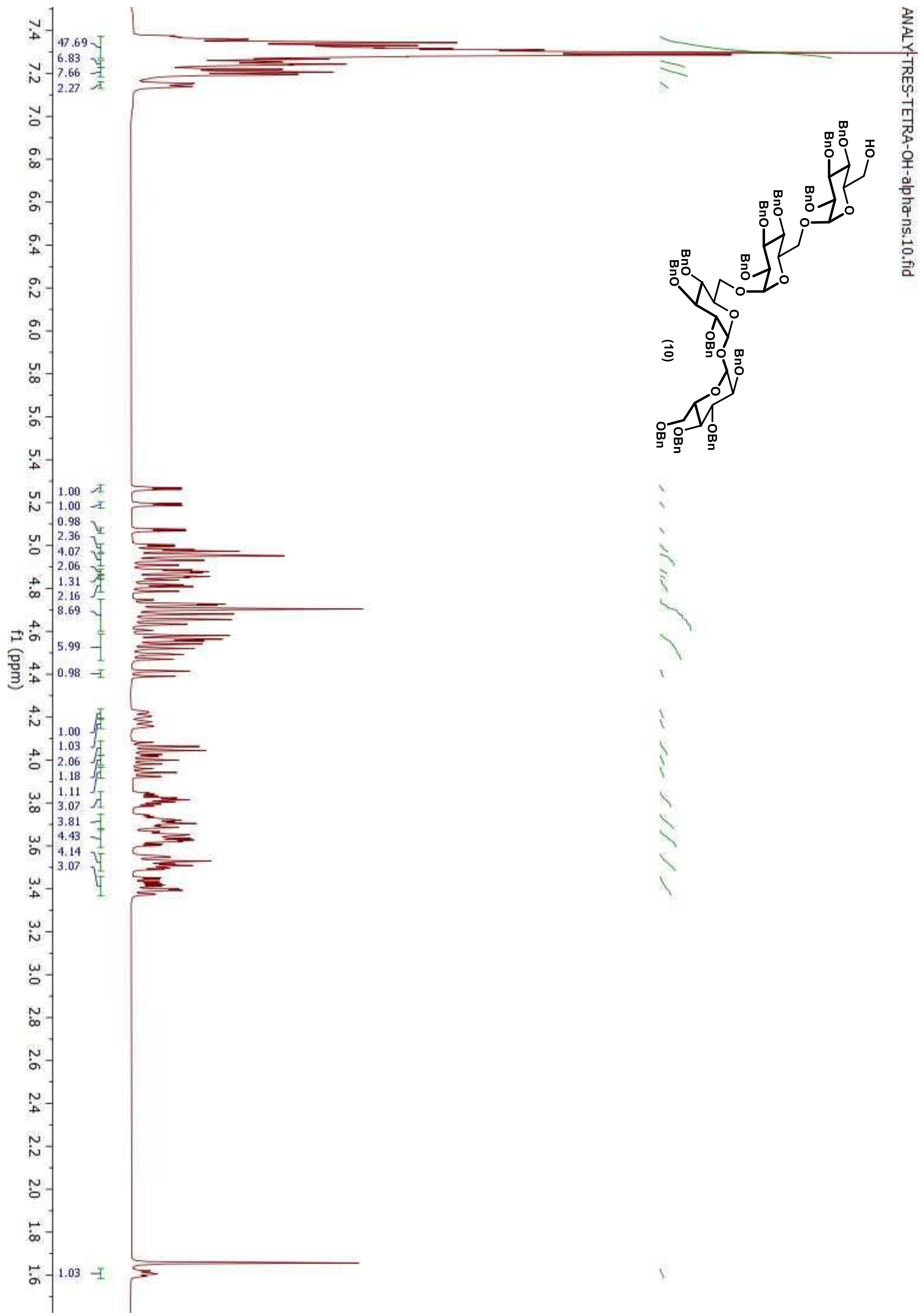
ANALY-TRES-TETRA-OH-alpha-ns.10.fid
(10)
f1 (ppm)

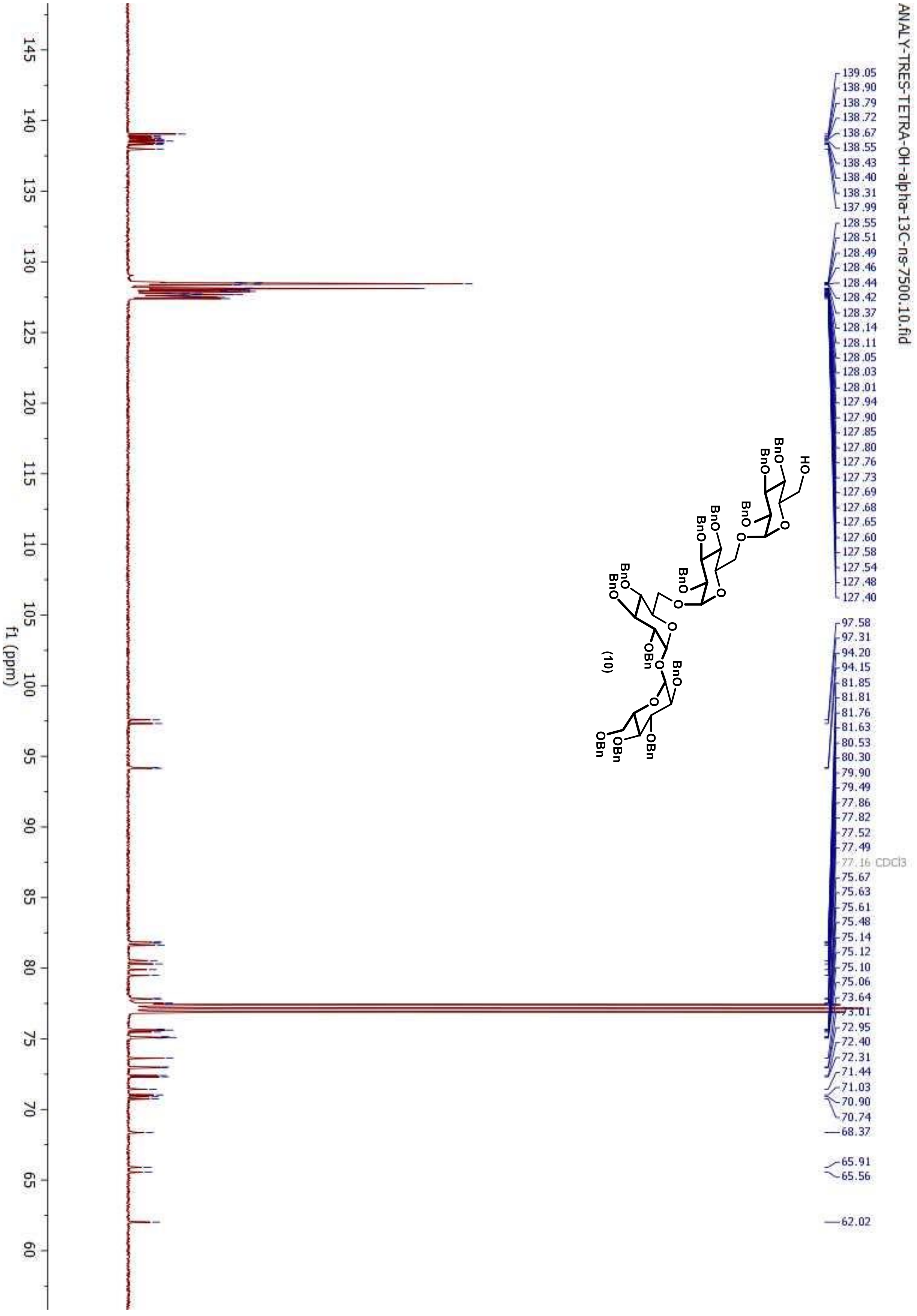

ANALY-TRES-TETRA-OH-alpha-13C-ns-7500.10.fid
139.05
138.90
138.79
138.72
138.67
138.55
138.43
138.40
138.31
137.99
128.55
128.51
128.49
128.46
128.44
128.42
128.37
128.14
128.11
128.05
128.03
128.01
127.94
127.90
127.85
127.80
127.76
127.73
127.69
127.68
127.65
127.60
127.58
127.54
127.48
127.40
97.58
97.31
94.20
94.15
81.85
81.81
81.76
81.63
80.53
80.30
79.90
79.49
77.86
77.82
77.52
77.49
77.16 CDCl3
75.67
75.63
75.61
75.48
75.14
75.12
75.10
75.06
73.64
73.01
72.95
72.40
72.31
71.44
71.03
70.90
70.74
68.37
65.91
65.56
62.02
HO
BnO
(10)
OBn
f1 (ppm)
145
140
135
130
125
120
115
110
105
100
95
90
85
80
75
70
65
60

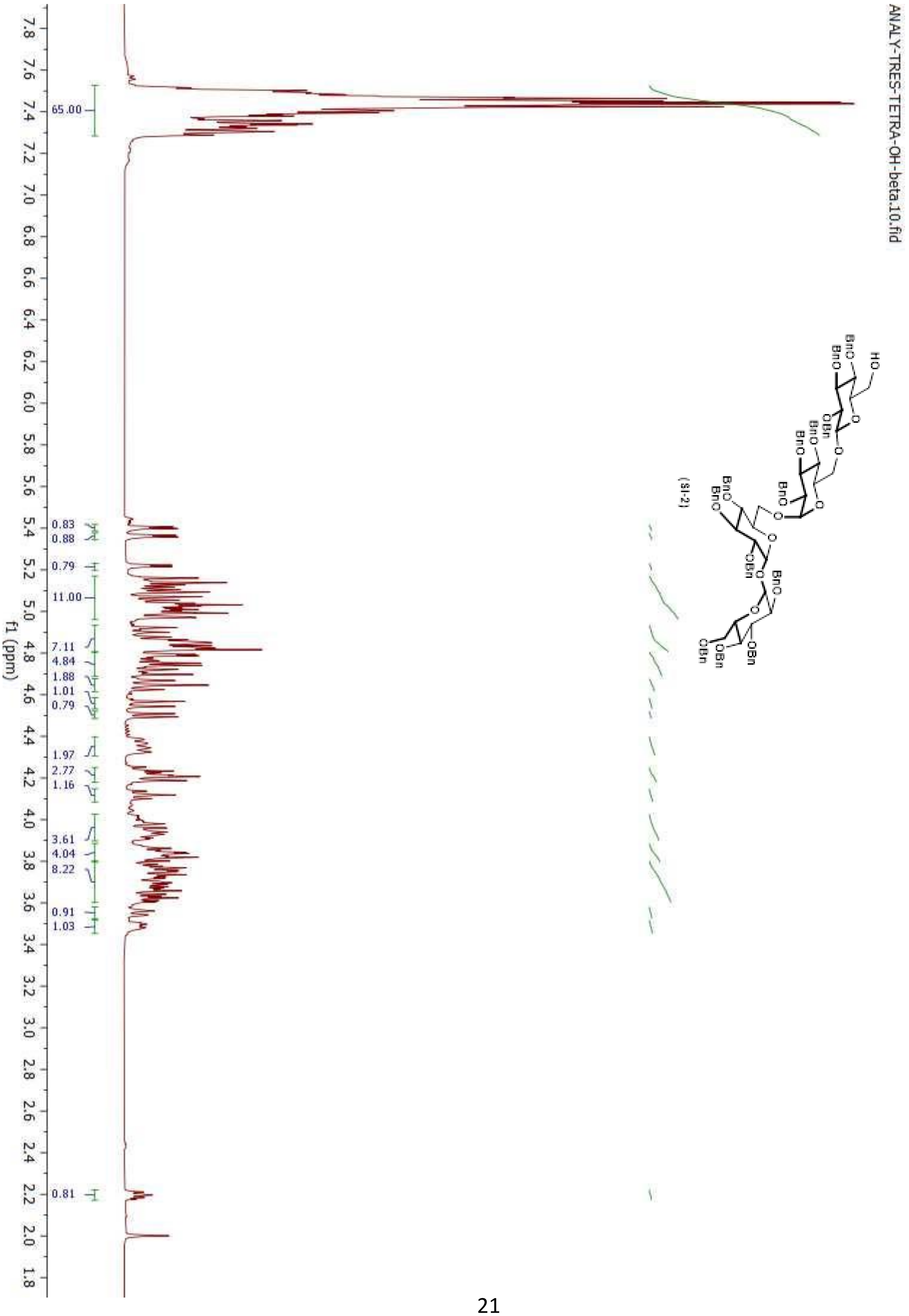
ANALY-TRES-TETRA-OH-beta.10.fid
(SI-2)
f1 (ppm)

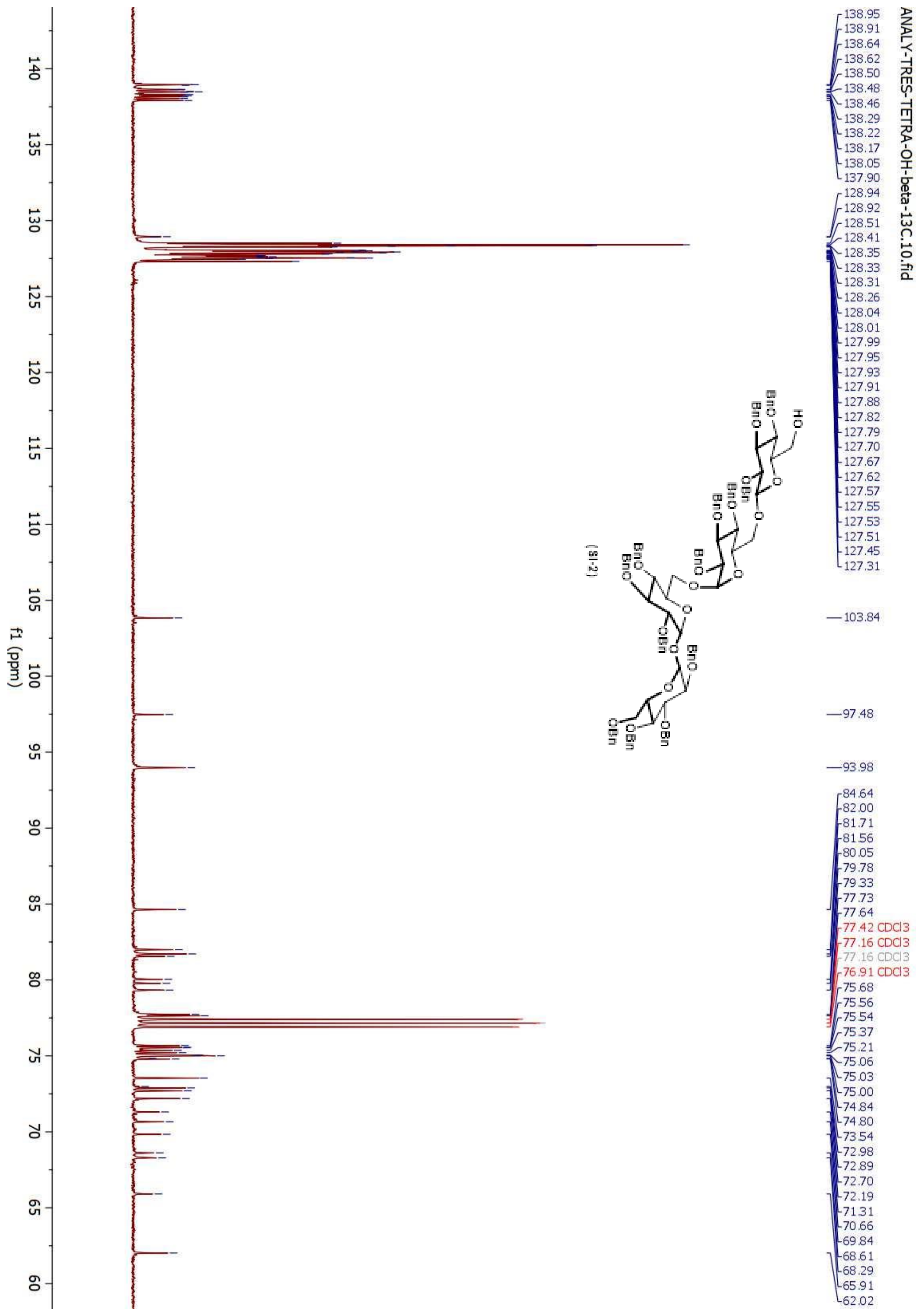
ANALY-TRES-TETRA-OH-beta-13C.10.fid
138.95
138.91
138.64
138.62
138.50
138.48
138.46
138.29
138.22
138.17
138.05
137.90
128.94
128.92
128.51
128.41
128.35
128.33
128.31
128.26
128.04
128.01
127.99
127.95
127.93
127.91
127.88
127.82
127.79
127.70
127.67
127.62
127.57
127.55
127.53
127.51
127.45
127.31
103.84
97.48
93.98
84.64
82.00
81.71
81.56
80.05
79.78
79.33
77.73
77.64
77.42 CDCl3
77.16 CDCl3
77.16 CDCl3
76.91 CDCl3
75.68
75.56
75.54
75.37
75.21
75.06
75.03
75.00
74.84
74.80
73.54
72.98
72.89
72.70
72.19
71.31
70.66
69.84
68.61
68.29
65.91
62.02
HO
BnO
BnO
OBn
BnO
BnO
BnO
BnO
BnO
OBn
BnO
OBn
OBn
OBn
(SI-2)
140
135
130
125
120
115
110
105
100
95
90
85
80
75
70
65
60
f1 (ppm)

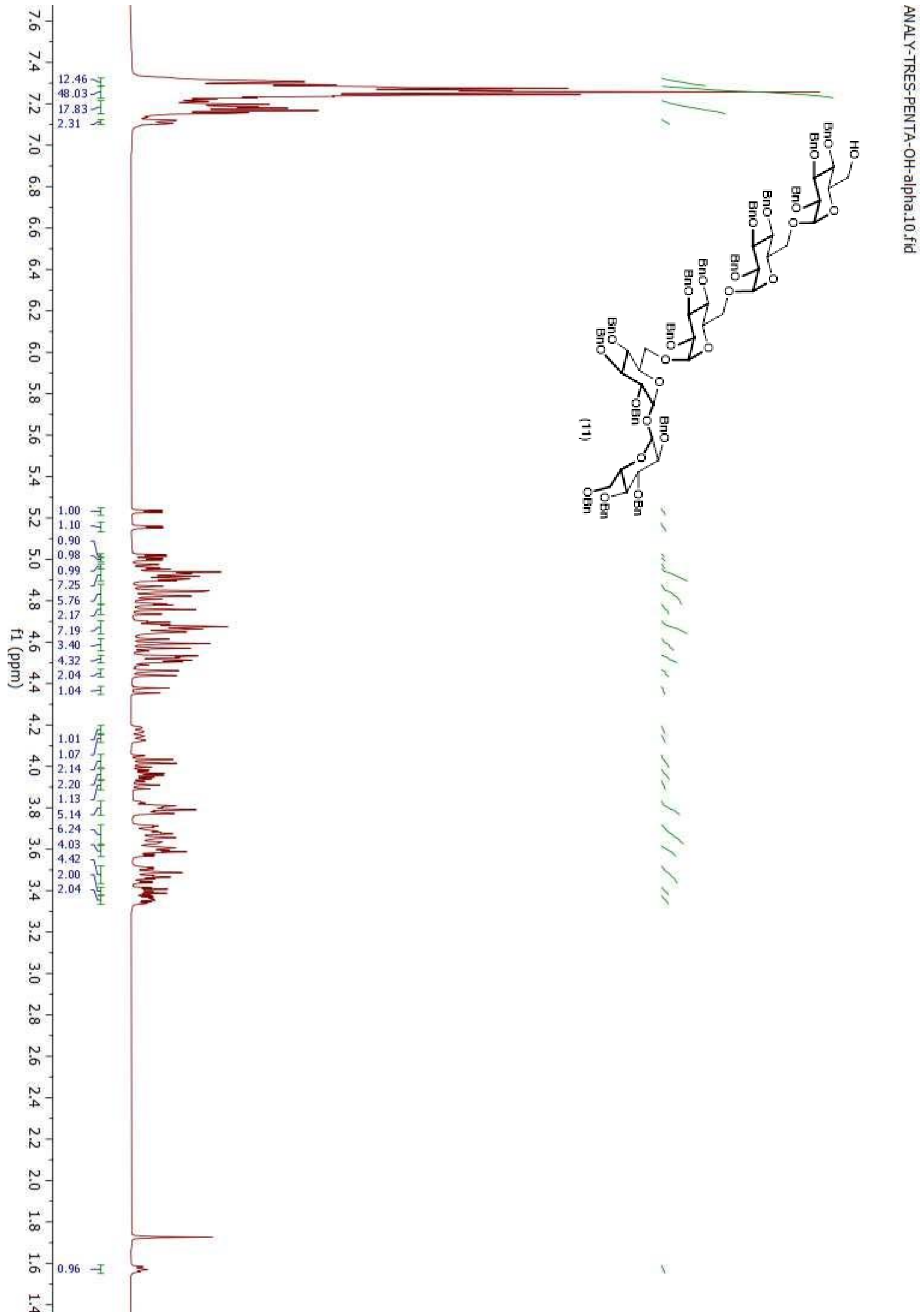
ANALY-TRES-PENTA-OH-alpha.10.fid
(11)
f1 (ppm)

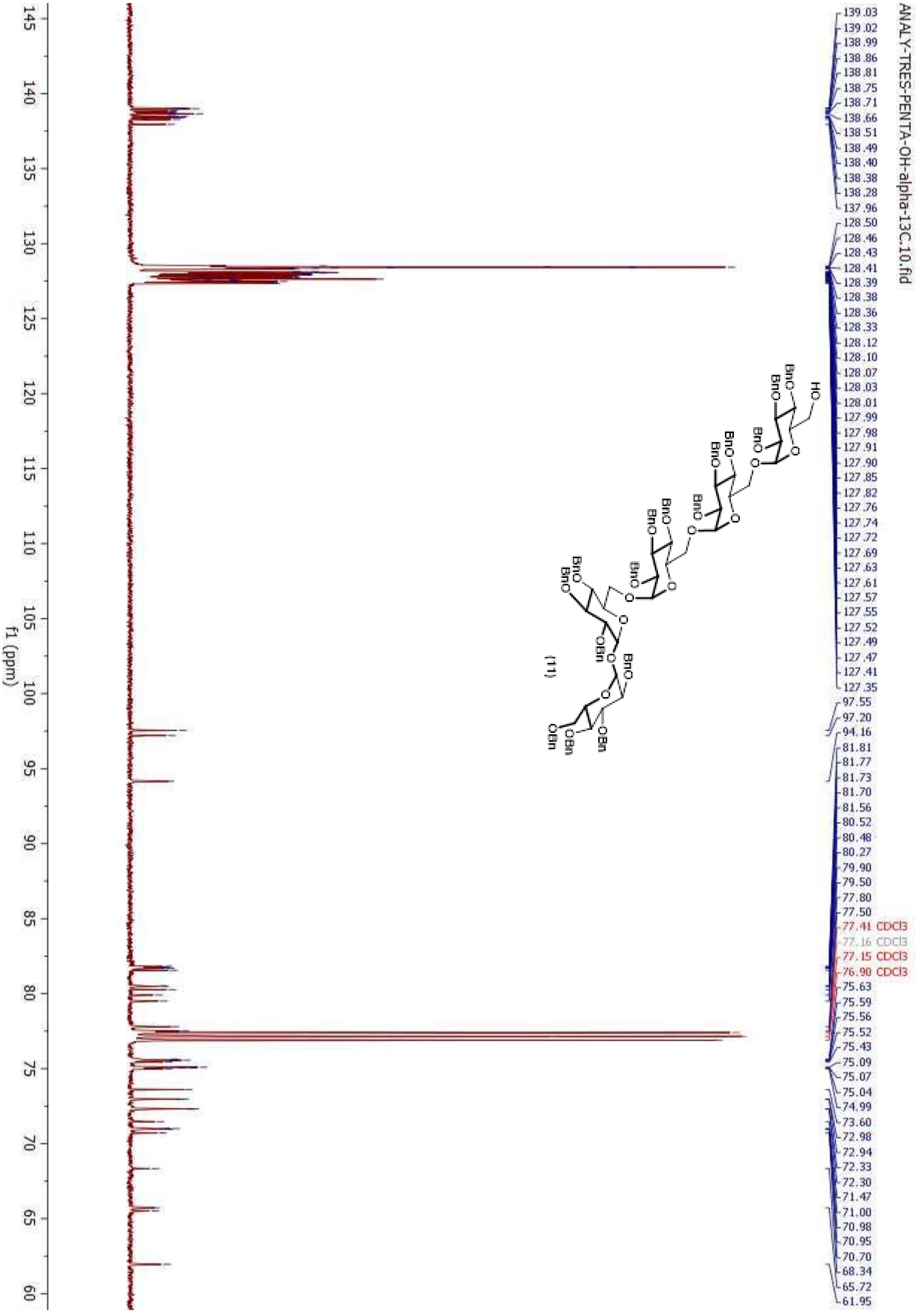

ANALY-TRES-PENTA-OH-alpha-13C.10.fid
139.03
139.02
138.99
138.86
138.81
138.75
138.71
138.66
138.51
138.49
138.40
138.38
138.28
137.96
128.50
128.46
128.43
128.41
128.39
128.38
128.36
128.33
128.12
128.10
128.07
128.03
128.01
127.99
127.98
127.91
127.90
127.85
127.82
127.76
127.74
127.72
127.69
127.63
127.61
127.57
127.55
127.52
127.49
127.47
127.41
127.35
97.55
97.20
94.16
81.81
81.77
81.73
81.70
81.56
80.52
80.48
80.27
79.90
79.50
77.80
77.50
77.41 CDCl3
77.16 CDCl3
77.15 CDCl3
76.90 CDCl3
75.63
75.59
75.56
75.52
75.43
75.09
75.07
75.04
74.99
73.60
72.98
72.94
72.33
72.30
71.47
71.00
70.98
70.95
70.70
68.34
65.72
61.95
HO
BnO
OBn
(11)
f1 (ppm)
145
140
135
130
125
120
115
110
105
100
95
90
85
80
75
70
65
60

ANALY-TRES-PENTA-OH-beta.10.fid

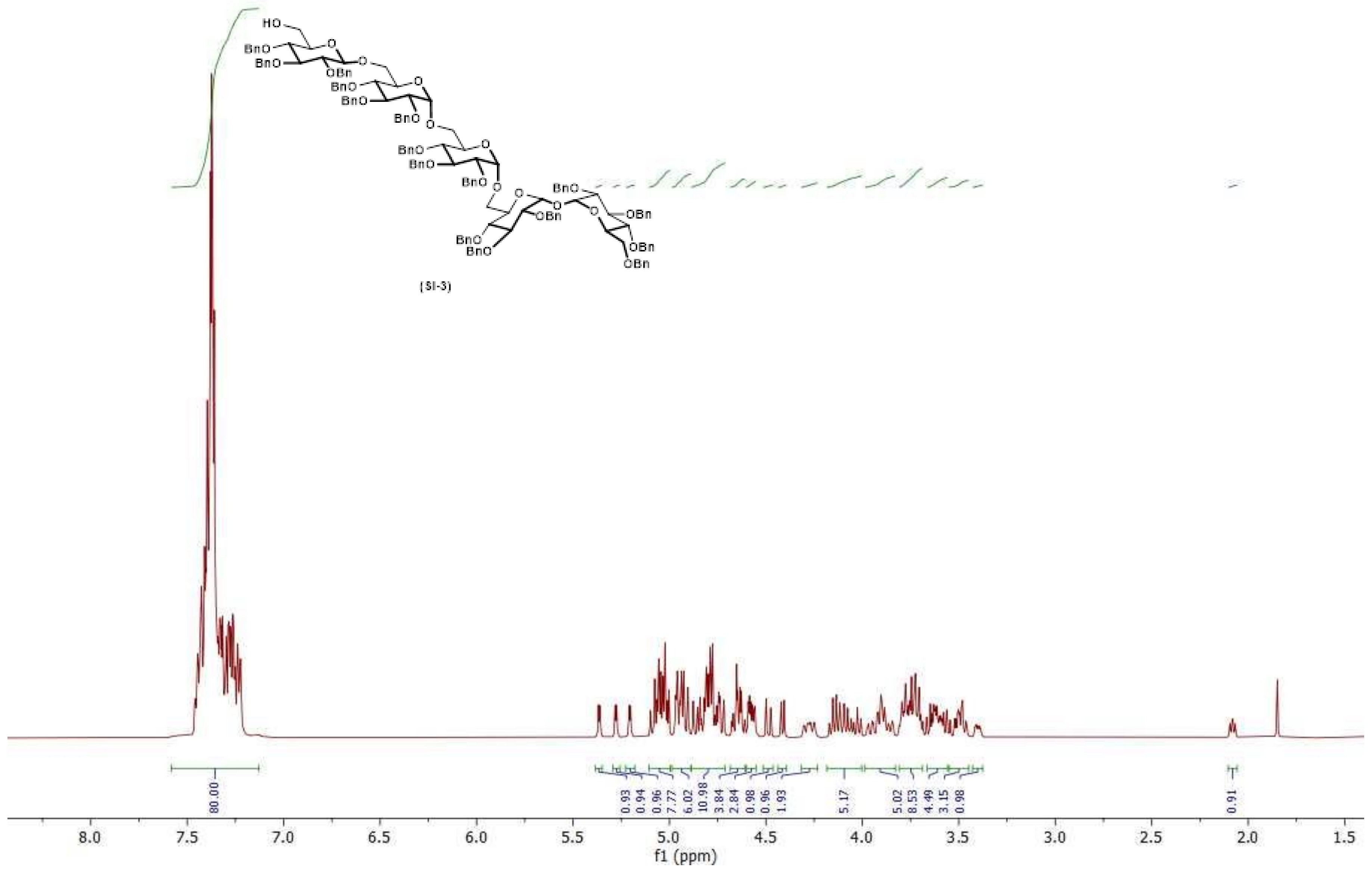

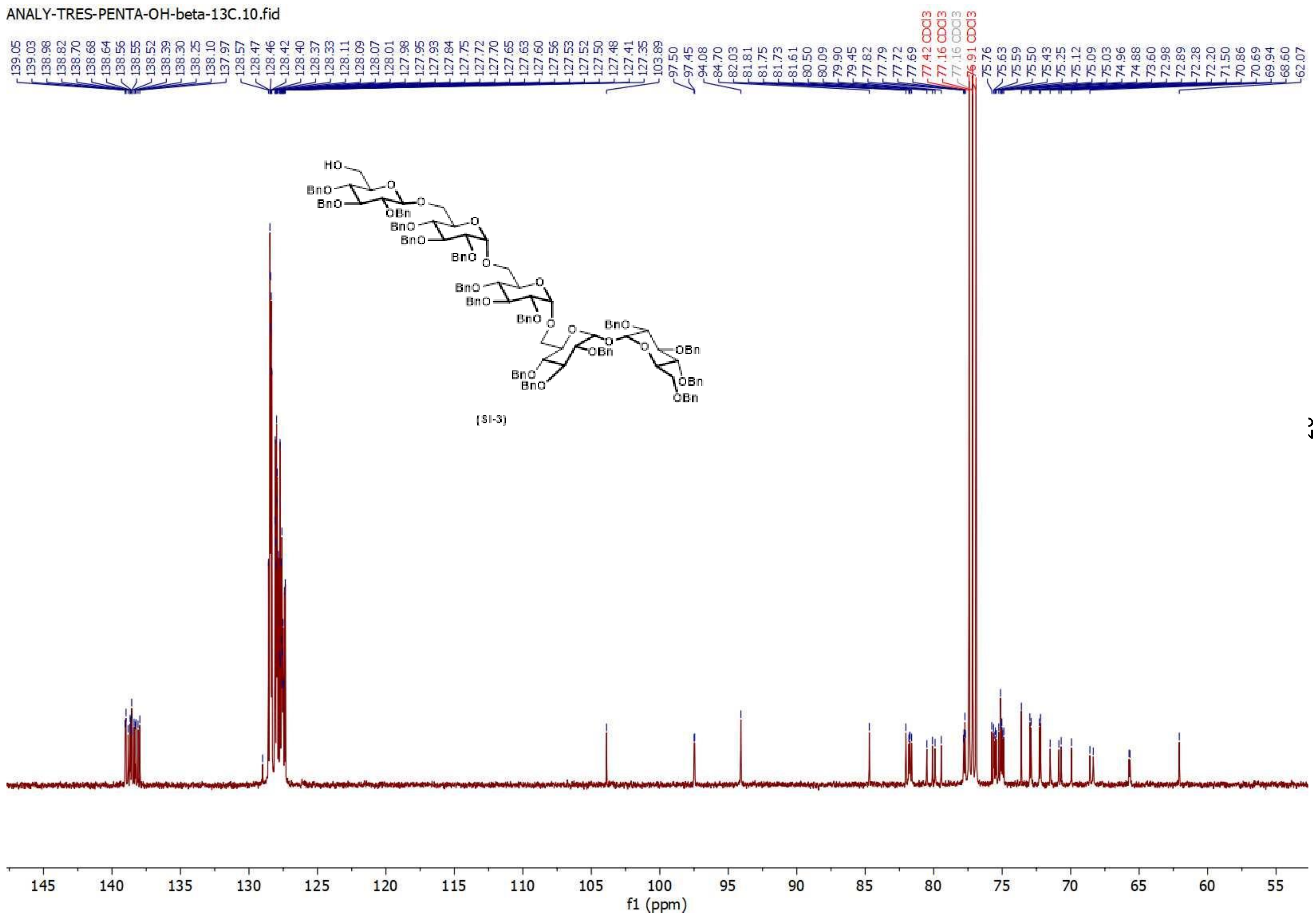
ANALY-TRES-PENTA-OH-beta-13C.10.fid
139.05
139.03
138.98
138.82
138.70
138.68
138.64
138.56
138.55
138.52
138.39
138.30
138.25
138.10
137.97
128.57
128.47
128.46
128.42
128.40
128.37
128.33
128.11
128.09
128.07
128.01
127.98
127.95
127.93
127.84
127.75
127.72
127.70
127.65
127.63
127.60
127.56
127.53
127.52
127.50
127.48
127.41
127.35
103.89
97.50
97.45
94.08
84.70
82.03
81.81
81.75
81.73
81.61
80.50
80.09
79.90
79.45
77.82
77.79
77.72
77.69
77.42 CDCl3
77.16 CDCl3
77.16 CDCl3
76.91 CDCl3
75.76
75.63
75.59
75.50
75.43
75.25
75.12
75.09
75.03
74.96
74.88
73.60
72.98
72.89
72.28
72.20
71.50
70.86
70.69
69.94
68.60
62.07
HO
BnO
OBn
O
(SI-3)
145
140
135
130
125
120
115
110
105
100
95
90
85
80
75
70
65
60
55
f1 (ppm)

ANALY-TRES-TRI-FINAL-alpha.10.fid

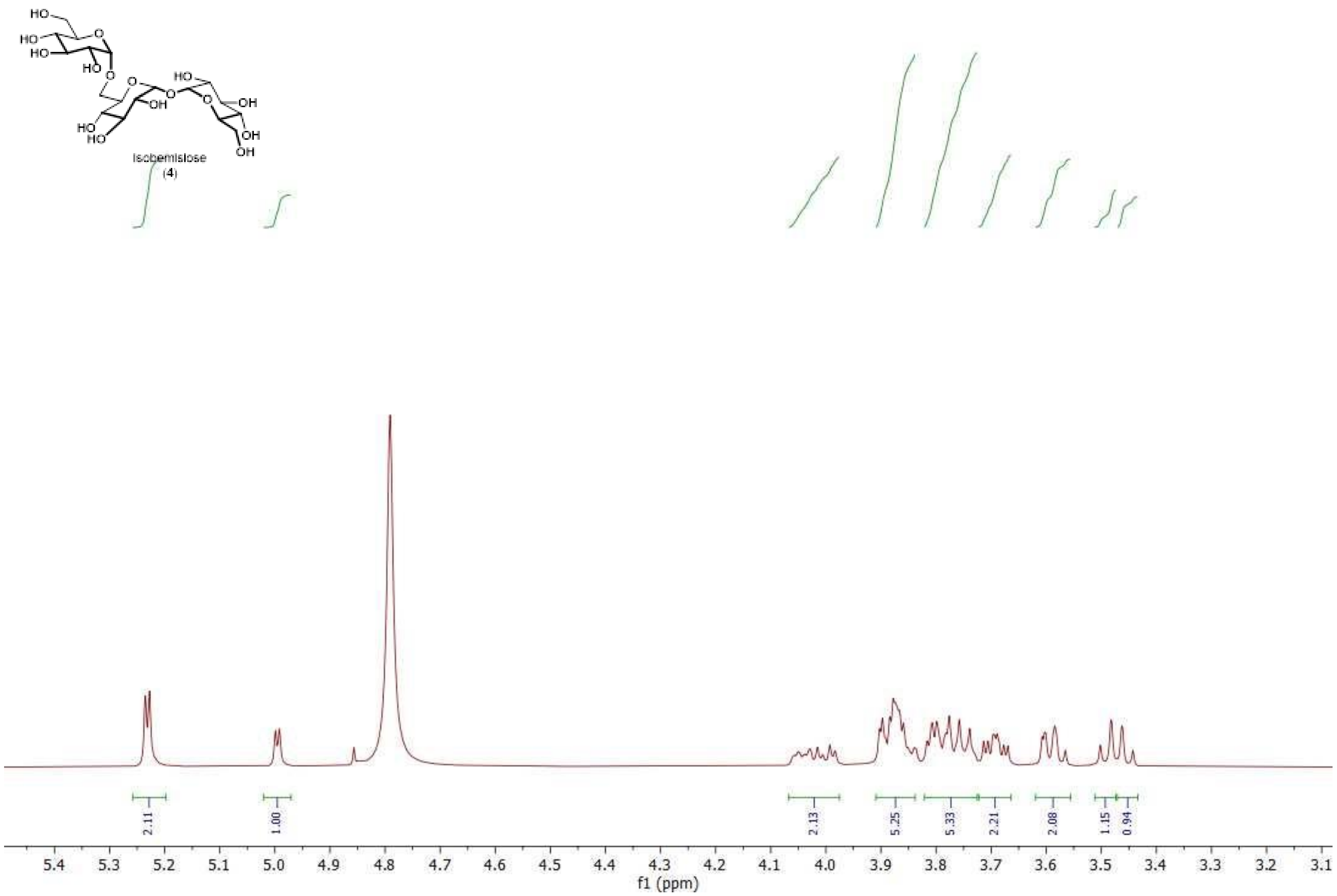

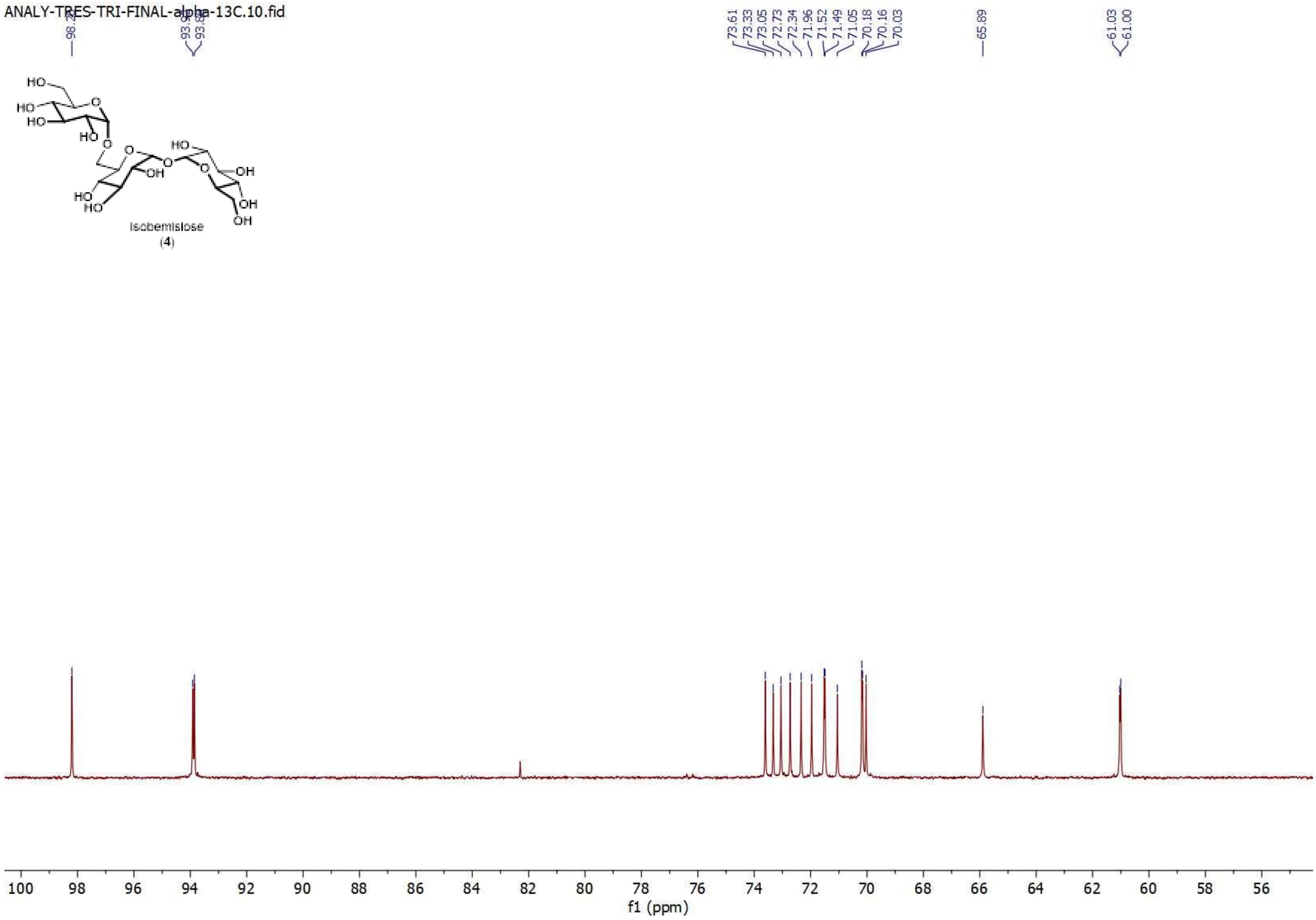
ANALY-TRES-TRI-FINAL-alpha-13C.10.fid
73.61
73.33
73.05
72.73
72.34
71.96
71.52
71.49
71.05
70.18
70.16
70.03
65.89
61.03
61.00
Isobemisiose
(4)
100 98 96 94 92 90 88 86 84 82 80 78 76 74 72 70 68 66 64 62 60 58 56
f1 (ppm)

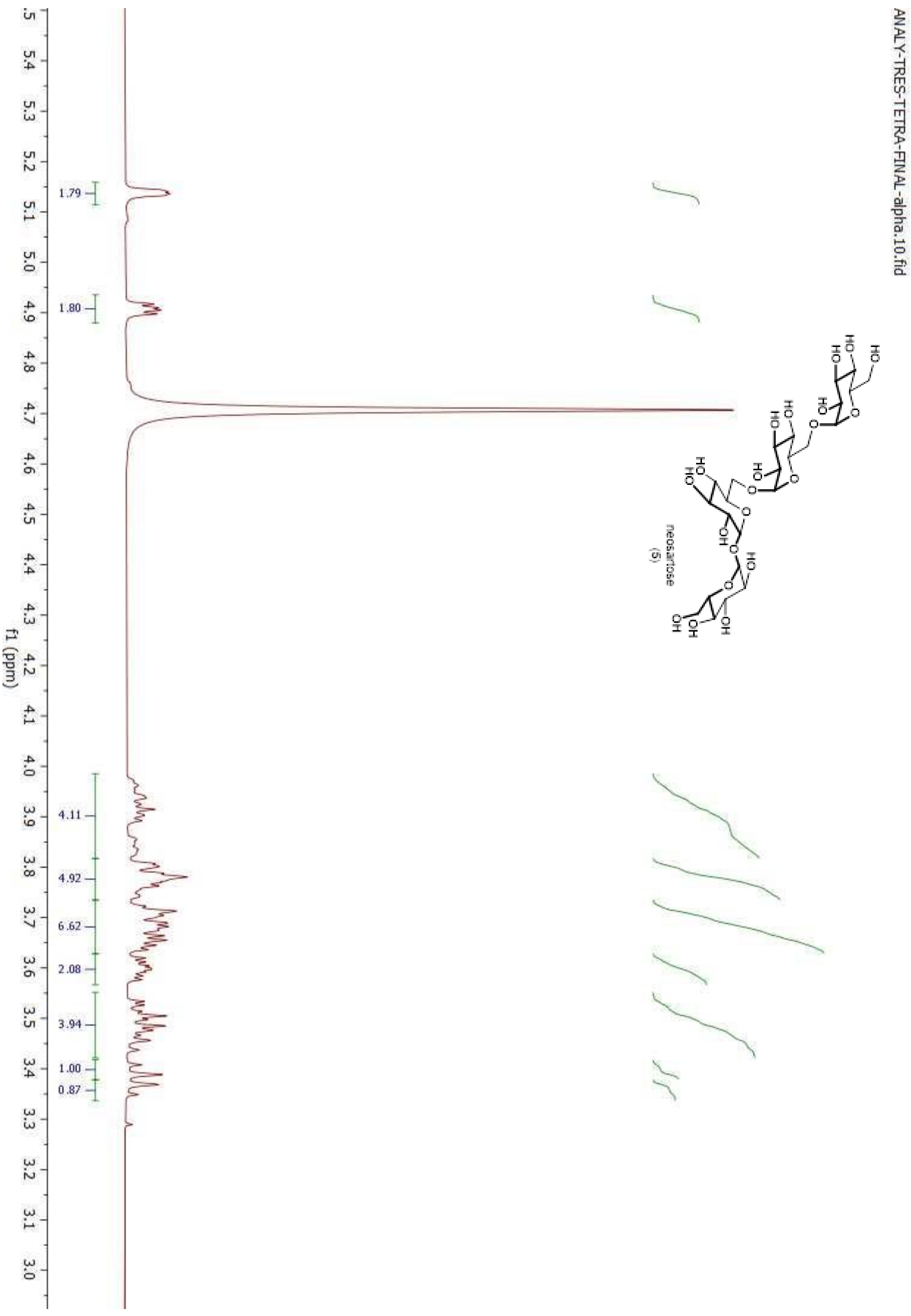
ANALY-TRES-TETRA-FINAL-alpha.10.fid
neosartose
(5)
1.79
1.80
4.11
4.92
6.62
2.08
3.94
1.00
0.87
5.4 5.3 5.2 5.1 5.0 4.9 4.8 4.7 4.6 4.5 4.4 4.3 4.2 4.1 4.0 3.9 3.8 3.7 3.6 3.5 3.4 3.3 3.2 3.1 3.0
f1 (ppm)

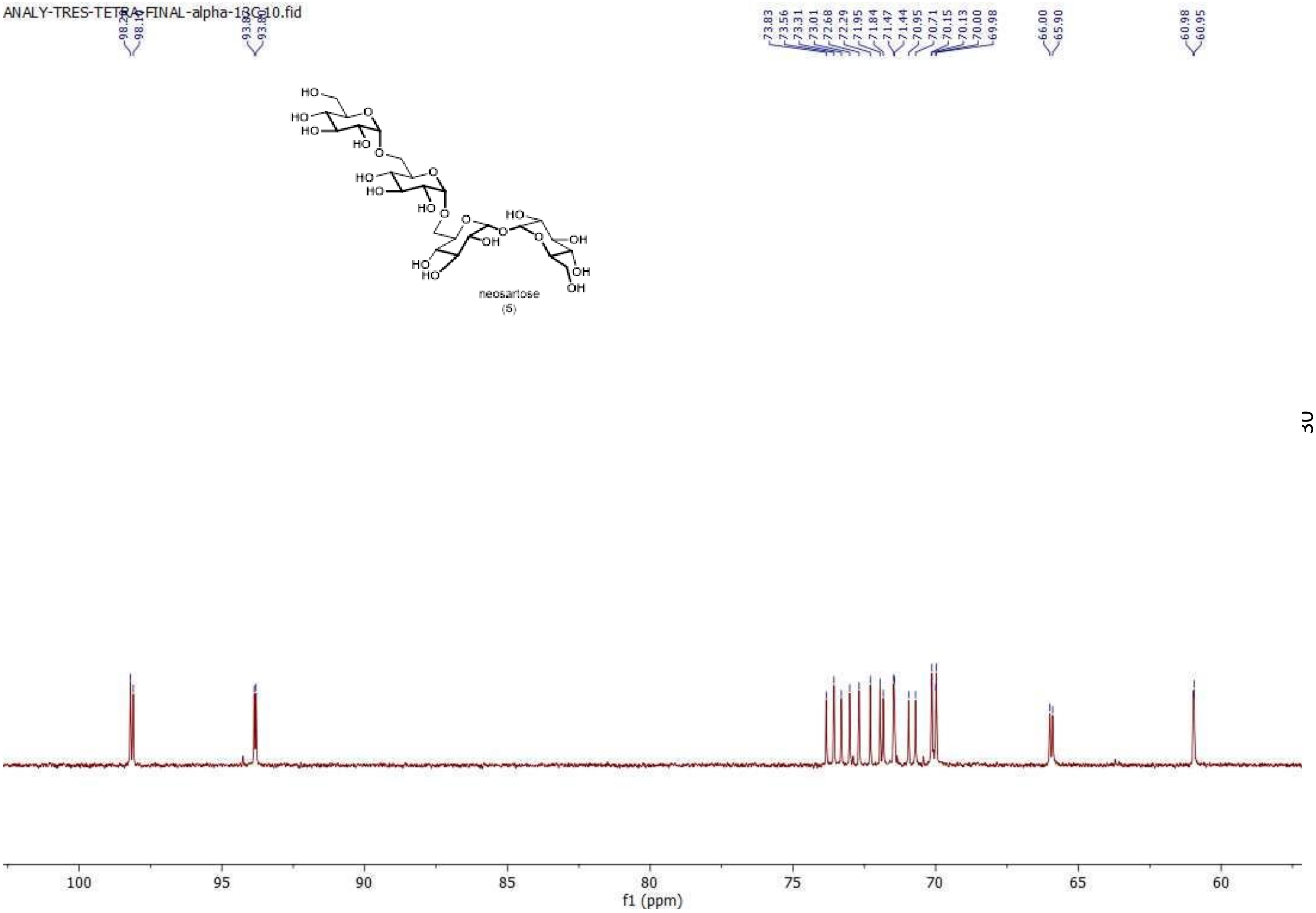
ANALY-TRES-TETRA-FINAL-alpha-13C-10.fid
neosartose
(5)
73.83
73.56
73.31
73.01
72.68
72.29
71.95
71.84
71.47
71.44
70.95
70.71
70.15
70.13
70.00
69.98
66.00
65.90
60.98
60.95
100
95
90
85
80
75
70
65
60
f1 (ppm)

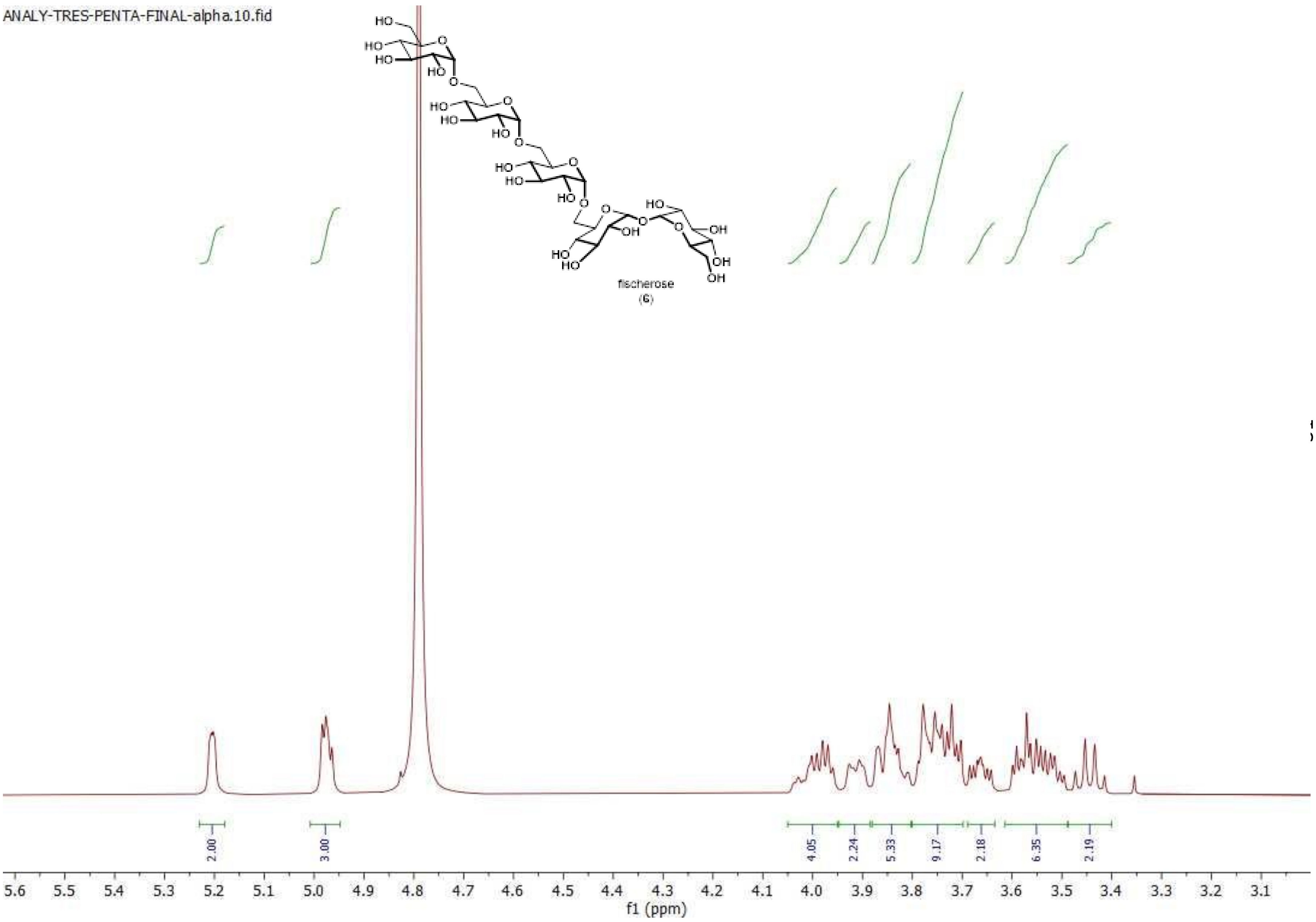
ANALY-TRES-PENTA-FINAL-alpha.10.fid
fischerose
(6)
2.00
3.00
4.05
2.24
5.33
9.17
2.18
6.35
2.19
5.6 5.5 5.4 5.3 5.2 5.1 5.0 4.9 4.8 4.7 4.6 4.5 4.4 4.3 4.2 4.1 4.0 3.9 3.8 3.7 3.6 3.5 3.4 3.3 3.2 3.1
f1 (ppm)

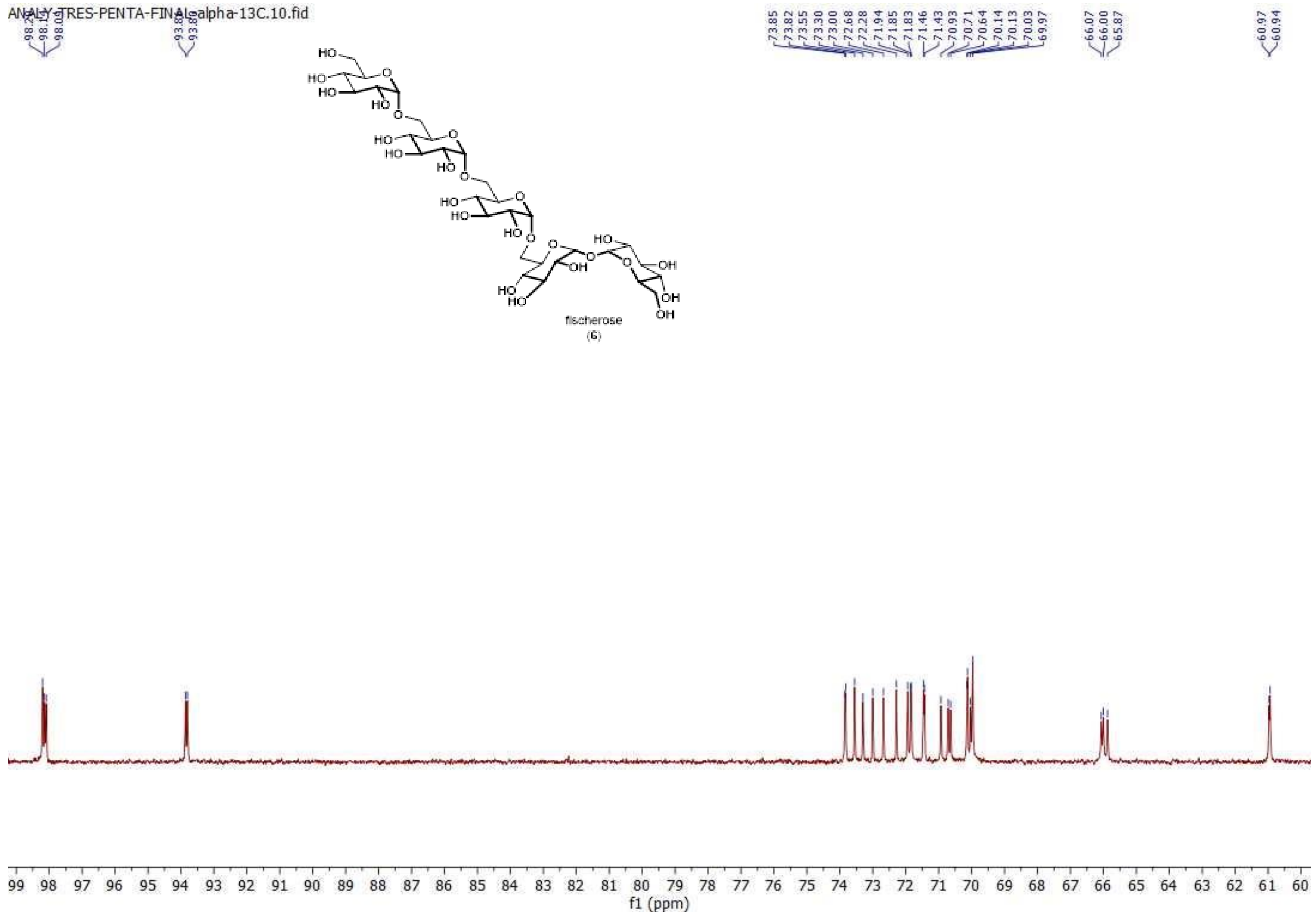
ANALY-TRES-PENTA-FINAL-alpha-13C.10.fid
73.85
73.82
73.55
73.30
73.00
72.68
72.28
71.94
71.85
71.83
71.46
71.43
70.93
70.71
70.64
70.14
70.13
70.03
69.97
66.07
66.00
65.87
60.97
60.94
fischerose
(6)
f1 (ppm)
